# Immobilization of Oxyanions on the Reconstructed Heterostructure Evolved from a Bimetallic Oxysulfide for the Promotion of Oxygen Evolution Reaction

**DOI:** 10.1007/s40820-023-01164-9

**Published:** 2023-07-29

**Authors:** Kai Yu, Hongyuan Yang, Hao Zhang, Hui Huang, Zhaowu Wang, Zhenhui Kang, Yang Liu, Prashanth W. Menezes, Ziliang Chen

**Affiliations:** 1 Institute of Functional Nano and Soft Materials (FUNSOM), Jiangsu Key Laboratory for Carbon-Based Functional Materials and Devices, Joint International Research Laboratory of Carbon-Based Functional Materials and Devices, Soochow University, Suzhou, 215123 People’s Republic of China; 2 Department of Chemistry: Metalorganics and Inorganic Materials, Technical University of Berlin, Straße Des 17 Juni 135. Sekr. C2, 10623 Berlin, Germany; 3 Materials Chemistry Group for Thin Film Catalysis – CatLab, Helmholtz-Zentrum Berlin für Materialien und Energie, Albert-Einstein-Str. 15, 12489 Berlin, Germany; 4 School of Physics and Engineering, Longmen Laboratory, Henan University of Science and Technology, Luoyang, 471023 People’s Republic of China

**Keywords:** Lanthanum-nickel oxysulfide, Rare earth metal, Immobilization of oxyanions, Structural reconstruction, Oxygen evolution catalysis

## Abstract

**Abstract:**

Efficient and durable oxygen evolution reaction (OER) requires the electrocatalyst to bear abundant active sites, optimized electronic structure as well as robust component and mechanical stability. Herein, a bimetallic lanthanum-nickel oxysulfide with rich oxygen vacancies based on the La_2_O_2_S prototype is fabricated as a binder-free precatalyst for alkaline OER. The combination of advanced in situ and ex situ characterizations with theoretical calculation uncovers the synergistic effect among La, Ni, O, and S species during OER, which assures the adsorption and stabilization of the oxyanion $\left( {{\text{SO}}_{4}^{{2 - }} } \right)$ onto the surface of the deeply reconstructed porous heterostructure composed of confining NiOOH nanodomains by La(OH)_3_ barrier. Such coupling, confinement, porosity and immobilization enable notable improvement in active site accessibility, phase stability, mass diffusion capability and the intrinsic Gibbs free energy of oxygen-containing intermediates. The optimized electrocatalyst delivers exceptional alkaline OER activity and durability, outperforming most of the Ni-based benchmark OER electrocatalysts.

**Graphical Abstract:**

**Supplementary Information:**

The online version contains supplementary material available at 10.1007/s40820-023-01164-9.

## Highlights

- A bimetallic lanthanum-nickel oxysulfide based on a La_2_O_2_S prototype was developed as a precatalyst for the electrochemical alkaline oxygen evolution reaction (OER).
- The in situ, ex situ, and theoretical investigations demonstrated that the precatalyst underwent a deep OER-driven reconstruction into a porous heterostructure where NiOOH nanodomains were uniformly separated and confined by La(OH)_3_ barrier.
- Oxyanion (SO_4_^2−^) was steadily adsorbed on the surface of this in situ reconstructed NiOOH/La(OH)_3_ heterostructure, enabling it for enhanced OER activity and durability.

**Supplementary Information:**

The online version contains supplementary material available at 10.1007/s40820-023-01164-9.

## Graphical abstract

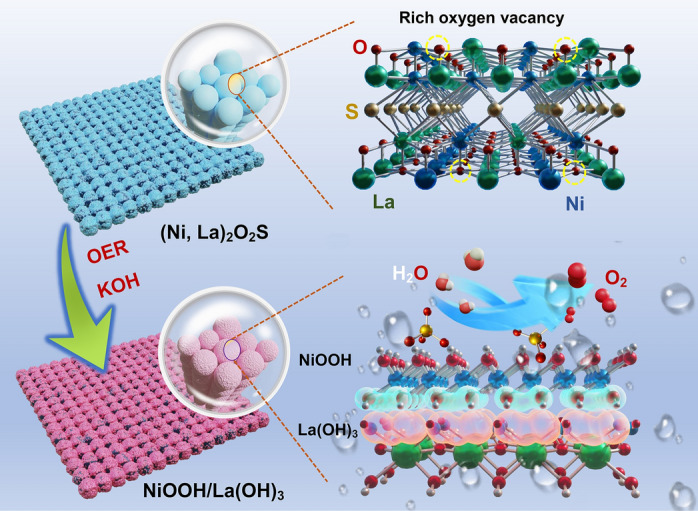

## Introduction

Electrocatalytic water splitting provides a sustainable pathway to produce hydrogen with high purity [1–7]. However, the sluggish kinetics in anodic oxygen evolution reaction (OER) severely degrades the efficiency of water electrolysis [8, 9]. To address this issue, noble metal-based oxides such as IrO_2_ and RuO_2_ are usually deployed as the OER catalysts to decrease the energy barrier, yet they suffer from high cost and extreme scarcity [10]. Within this context, the research community has recently focused their attention on low-cost transition metal (TM)-based compounds (e.g., alloys, intermetallics, (hydro)oxides, sulfides, phosphides, and borides), and is committed to developing them into high-performance OER electrocatalysts [11–15]. Despite the great progress that has been achieved in recent years, it is still of great interest to explore novel catalytic systems with high activity and long durability as well as understand the correlation between their microstructure and performance.

Current research on the TM-based alkaline OER electrocatalysts concentrates on the composition composed of TM and anions (e.g., B, P, S, Se, Te), metalloids (e.g., Si, Ge, As) or lean metals (e.g., Al, Ga, Sn) [14–26]. Intriguingly, these compounds are basically deemed as the precatalysts, in which the TM species convert into active TM (oxy)hydroxides while the nonmetals mostly leach out of the structure during alkaline OER, leading to the formation of active nanodomains and thereby improving catalytic activity [18, 22, 26]. Based on this premise, it could be naturally envisioned that to achieve the high activity, enormous active nanodomains must be created by phase reconstruction during OER. Since the phase reconstruction degree is highly dependent on the particle size of precatalysts, nanosizing has been demonstrated as a powerful strategy to enable deep phase reconstruction [19, 27, 28]. Although the degree for the phase reconstruction can be readily promoted by the nanosizing approach, the generated active nanodomains would gradually aggregate under the long-term OER due to their high surface energy, resulting in the blocking of accessible active sites and thus, decreasing the activity [19]. An effective phase reconstruction should not only allow the deep transformation of the precatalysts but also should have the ability to avoid the agglomeration of active nanodomains. Therefore, to be the ideal precatalyst, a material should satisfy the following requirements: (1) it should present in the form of nanosize, which allows the binding of sufficient (oxy)hydroxide ligands with metal sites [19, 28]; (2) some anions such as sulfur or phosphorus species which possess the high thermodynamics destabilization tendency during alkaline OER should be contained in the precatalysts, the leaching of which would generate the pores and synergistically contribute to the deep transformation [13, 29]; (3) some catalytically inactive subunit should concurrently be formed during the phase reconstruction, which then serves as the fence to in situ confine the active nanodomains to suppress the agglomeration [19, 22]. To the best of our knowledge, such a pristine precatalyst that integrates these three merits has never been perceived.

On the other hand, previous studies also claimed that the oxyanions species (such as SO_4_^2−^, SeO_3_^2−^, PO_4_^2−^) formed during the reconstruction of precatalysts were expected to be adsorbed onto the surface of TM (oxy)hydroxides, which then modulated the electronic structure of active sites and thereby an improvement of the intrinsic OER activity [13, 30, 31]. Generally, the oxygen atoms-exposed surfaces are deemed as the active surface of TM (oxy)hydroxides [32, 33], but which probably bear the intrinsically weak adsorption ability toward the oxyanions due to the negative charge repulsion, and thus lead to the detachment of the oxyanions from the active surface as the OER progresses and finally degrade the activity and durability. Thus, it is of great interest to explore the feasible strategy to immobilize the oxyanion species on the surface of reconstructed TM (oxy)hydroxides during OER. However, rare or not much attention has been paid to the related investigation.

In view of the above considerations, lanthanum oxysulfide (La_2_O_2_S) was deliberately selected as the host to design the well-defined OER precatalysts because of its following potential features [13, 19, 22, 33–42]: (1) Oxygen vacancies exist in the host structure, which is believed to promote the oxidation reaction during alkaline water splitting; (2) Sulfur anion species tends to leach from the structure, resulting in the porous structure; (3) *f*-block rare earth metals (exemplified by La) have been confirmed to play pivotal roles in catalysis including water oxidation. First of all, different from the easy leaching of *s*- and *p*-block elements, rare earth metals are prone to form catalytically inactive precipitate (oxy)hydroxides during alkaline OER, serving as the effective barrier suppressing the agglomeration of active sites [19, 33, 36, 43]. Meanwhile, the in situ formed rare earth metal (oxy)hydroxides during OER can provide a significant contribution toward electronic interaction with the active TM (oxy)hydroxides, regulating its electronic structure and optimizing the adsorption capacity toward the reaction intermediates [19, 33]. Additionally, the formation of rare earth metal (4f) ─ O (2p) ─ TM (3d) by orbital coupling could promote the covalency of TM (3d) ─ O (2p) with more and faster electrons injection, which can optimize the interaction between TM sites and O-containing intermediates [22, 44–46]. Besides, the presence of oxidized rare earth metal species could substantially prevent the severe loss of the active TM atoms, elevating the catalytic stability of the active TM-based structures during OER [47, 48]; (4) The crystal structure of La_2_O_2_S can well accommodate the incorporation of heterometal atoms to the La-site in a wide composition range, offering the chance to incorporate the active TM sites. Meanwhile, given the fact that Ni species is one of the most widely used ones for the alkaline OER, in this contribution, the Ni-incorporated La_2_O_2_S precatalyst is controllably synthesized as an OER precatalyst through the sequential hydrothermal-annealing strategy. As expected, the optimum Ni-incorporated La_2_O_2_S (NLOS-1) exhibited an outstanding water oxidation activity in 1 M KOH media affording 50 mA cm^−2^ at an ultralow overpotential of about 260 mV and also maintained a high current density of 100 mA cm^−2^ over three days with negligible activity decay. Such an OER performance was significantly superior to that of bare nickel sulfide precatalyst under identical test conditions and even better than those of most recently documented Ni-based benchmark OER precatalysts. Furthermore, a combination of various ex/in situ characterizations and theoretic density functional theory (DFT) calculations revealed that the incorporation of Ni cannot only effectively decrease the particle size, but also introduces more oxygen vacancies, which in turn induce more exposure of surface metal sites and accelerate interfacial charge transfer kinetics. Benefiting from these merits as well as the synergistic effect of all components in NLOS, its surface was deeply reconstructed into porous NiOOH/La(OH)_3_ heterostructure accompanied by the loss of sulfur species, where highly dispersive ultrasmall NiOOH nanodomains were clearly separated by numerous inactive La(OH)_3_ in a pseudo-periodic arrangement. This ensured the sufficient exposure and accessibility of active sites of NiOOH surface, as well as promoted effective mass transport during OER. More importantly, this heterostructure could readily adsorb and stabilize the SO_4_^2−^ anions against OER, which not only remarkably regulated the electronic structure of NiOOH, improving its inherent adsorption-free energy toward OER intermediates, but also effectively maintained the high activity for the long run.

## Experimental Section

### Preparation of NiLa-X@CC and Ni LDH@CC

Prior to all synthesis, the fresh CC was treated to remove its surface impurities by washing in acetone, ethanol, and deionized water as well as subsequently activated and hydrophilized in the nitric acid solution for 24 h at 90 °C under a continuous stirring. To illustrate the preparation of CC-supporting NiLa hydroxide precursors with a different molar ratio of Ni and La (NiLa-X@CC, *X* = 0, 0.25, 0.5, 1, 2, respectively), the one with a ratio of “1:1” was exemplified in details: 433 mg La(NO_3_)_3_·6H_2_O, 290 mg Ni(NO_3_)_2_·6H_2_O, and 280 mg hexamethylenetetramine were mixed into 20 mL methanol under a vigorous magnetic stirring until a homogeneous solution was formed. Then, the pretreated CC was immersed into the above mixture and transferred to a 50 mL Teflon-lined stainless-steel autoclave. After being well sealed, the autoclave was maintained at 160 °C for 10 h, which was then cooled down to room temperature naturally. The as-prepared NiLa-X@CC sample was obtained after washing with deionized water and ethanol several times and drying in a vacuum. For “Ni:La = 0:1”, “Ni:La = 1:4”, “Ni:La = 1:2”, and “Ni:La = 2:1” samples, the same procedure was adapted except for the molar ratios of the utilized Ni and La source were accordingly modified. In addition, we also synthesized the pure Ni LDH@CC precursor following the same fabricating conditions for NiLa-X@CC, except no La source was added.

### Preparation of NLOS-X@CC and Ni_3_S_2_@CC

20 mg sublimed sulfur and a piece of carbon cloth deposited by NiLa-X@CC precursor were placed at the upstream and downstream side of the tube furnace, respectively, followed by being heated up to 600 °C for 2 h with a ramp rate of 5 ℃ min^−1^ under Ar atmosphere. The as-obtained Ni-incorporated La_2_O_2_S-X@CC were denoted as NLOS-X@CC, where “X” meant the molar ratio of Ni and La source, i.e., 0, 0.25, 0.5, 1, 2, respectively. On the other hand, the Ni LDH@CC precursor was thermally treated by using the same procedure, and the Ni_3_S_2_@CC product was successfully obtained.

### Characterizations

Powder X-ray diffraction (XRD) (Philips X’pert PRO MPD diffractometer, Holland) with Cu Kα radiation source (*λ* = 0.15406 nm) was used to characterize the crystalline phase information of the investigated samples. The morphology and surface structure of all the involved samples were characterized by field emission scanning electron microscopy (FESEM) (ZEISS G500) and transmission electron microscopy (TEM) (Talos F200X). To gain insights into the chemical state of the probed samples, X-ray photoelectron spectroscopy (XPS) was carried out at a Thermo Scientific K-Alpha spectrometer using Al *Kα* X-ray source (1486 eV). The electron paramagnetic resonance (EPR) spectra were obtained by a Bruke EMP plus EPR spectrometer (Room temperature, X band). To investigate the phase evolution, the in situ Raman spectra were collected through a Renishaw Raman system (in Via Qontorin, 532 nm laser) integrated with an electrochemical workstation (CHI, 760E). In addition to the two spectra recorded at the open circuit potential (OCP) before and after the water oxidation reaction, the applied potential increased from 1.0 to 1.8 V (vs. reversible hydrogen electrode, RHE) at a stepwise of 0.1 V. To further understand the specific oxidation state and local atomic structure of our target catalyst before and after OER process, La XAS measurements at *L*_3_ edge were performed on the BL14W beamline at the Shanghai Synchrotron Radiation Facility (SSRF). The elemental contents of the samples were determined by Inductively coupled plasma-optical emission spectra (ICP-OES) tests (Varian 720-ES) and energy-dispersive X-ray spectroscopy (EDS) characterizations coupled with the TEM.

### Electrochemical Measurements

Electrochemical measurements were operated in a standard three-electrode system in 1 M KOH using a CHI 760E electrochemistry workstation. The synthesized samples were used as working electrodes. A Hg/HgO electrode and a graphite rod were selected as the reference electrode and counter electrode, respectively. The loading mass of the probed samples on CC was around 3 mg cm^−2^ with a geometric area of 0.25 cm^−2^. To prepare nickel foam-supported NLOS-1 (NLOS-1@NF), a spraying method was employed to deposit the catalyst ink (mixed with 600 μL ethanol, 300 μL H_2_O, and 100 μL Nafion) on NF. The loading mass was around 4 mg cm^−2^. The linear sweep voltammetry (LSV) for OER was conducted at a scan rate of 5 mV s^−1^ with an applied IR compensation of 90%. In order to normalize the potentials against Hg/HgO to the values against RHE, a calibration equation was obtained experimentally according to the previous report [49]. In detail, using a sealed three-electrode system where Pt plates served as both working and counter electrodes, the CV curves were run at the scan rate of 1 mV s^–1^ in 1 M KOH saturated with hydrogen. The average of the two potentials corresponding to the zero-crossing current was regarded as the potential value referred to RHE. As a result, the calibration equation was determined as *E*_vs. RHE_ = *E*_vs. Hg/HgO_ + 0.930 V. The Tafel slope was determined on the basis of the equation: *η* = blog *j* + *a*, where *η*, *b*, and *j* represent overpotential (V), Tafel slope (mV dec^−1^), and current density (mA cm^−2^), respectively. The cyclic voltammetry (CV) measurements were operated at a non-Faradaic voltage region (0.73–0.93 V vs. RHE) to obtain the electrochemical double-layer capacitance (*C*_dl_). We plotted half of the difference for current densities at the middle potential of the CV window as a function of the associated scan rate, which can yield the corresponding slope as the specific value of *C*_dl_. Because electrochemical active surface area (ECSA) is proportional to *C*_dl_ (ECSA = *C*_dl_/*C*_s_, where the *C*_s_ is the specific capacitance of the material per unit area under identical electrolyte conditions), and *C*_s_ is assumed to be identical for all probed samples in our case, we directly normalized the current densities of LSV curves against the corresponding *C*_dl_ values to show the intrinsic activity [26, 29]. The Electrochemical impedance spectroscopy (EIS) was measured at a frequency starting from 100 kHz to 0.01 Hz. The chronopotentiometry (CP) test was carried out to estimate the OER stability.

### Transient Photo-Induced Voltage (TPV) Principle

The stimulus response method called TPV measurement was carried out on a self-made measurement system, in which the powder sample (obtained by the same procedure of NLOS-X@CC, except no CC substrate was added) covering platinum mesh (1 cm × 1 cm) was used as the working electrode and the platinum wire was used as the counter electrode. All measurements were performed at room temperature. The laser pulse (*λ* = 355 nm, pulse width 5 ns) was generated by the third harmonic Nd: YAG laser (Polaris II, New Wave Research, Inc.) irradiated on the powder sample. The generated photocurrent was first identified and amplified, and then the oscilloscope recorded the photocurrent as the ratio of the photovoltage to the internal resistance of the test system.

### Theoretic Calculations

DFT was carried out for all theoretical calculations, during which the Vienna ab initio Simulation Package (VASP) code was employed [50, 51]. The electron–ion interaction was adopted in the projector augmented wave method [52]. The electron exchange and correlation energy were described within the generalized gradient approximation in the Perdew–Burke–Ernzerhof formalism [53]. The NiOOH/La(OH)_3_ heterojunction was built by a (√5 × √5) La(OH)_3_ supercell along the (001) facet and a (4 × 4) NiOOH supercell along the (001) facet and the thickness of the vacuum was set as 16 Å. This supercell contains 12 La atoms, 32 Ni atoms, 68 H atoms, and 100 O atoms. The lattice mismatch of this supercell was smaller than 2%. The (001) facet is selected here because of the fact that it is the most stable surface in NiOOH for OER catalysis [32], and to better build the heterointerface by using these two complex hexagonal-type phases, the La(OH)_3_ (001) layer was chosen to match with NiOOH (001) layer. All atoms in heterojunction were relaxed for structural optimization. For the sampling of Brillouin-zone integrals, Gamma centered *k*-points grid of 2 × 2 × 1 was deployed [54]. The valence electrons are expanded in a plane-wave basis set with an energy cutoff of 450.0 eV. The convergence criteria of force and energy were 0.02 eV Å^−1^ and 10^−4^ eV, respectively.

During water oxidation, the free energy differences of each step (∆*G*) can be theoretically calculated using the following equations [33, 55]:

$$\Delta G_{{1}} = G\left( {*{\text{OH}}} \right){-}G\left( * \right){-}\mu {\text{OH}} = E\left( {*{\text{OH}}} \right){-}E\left( * \right){-}E\left( {{\text{H}}_{{2}} {\text{O}}} \right) + {1}/{2}E\left( {{\text{H}}_{{2}} } \right){-}eU + \Delta G_{{\text{H}}} + \left( {{\text{pH}}} \right) + \Delta \left( {{\text{ZPE}}{-}T\Delta S} \right)$$

$$\Delta G_{{2}} = G\left( {*{\text{O}}} \right){-}G\left( {*{\text{OH}}} \right) + \mu {\text{H}} = E\left( {*{\text{O}}} \right){-}E\left( {*{\text{OH}}} \right){-}E\left( {{\text{H}}_{{2}} {\text{O}}} \right) + {1}/{2}E\left( {{\text{H}}_{{2}} } \right){-}eU \, + \Delta G_{{\text{H}}} + \left( {{\text{pH}}} \right) + \Delta \left( {{\text{ZPE}}{-}T\Delta S} \right)$$

$$\Delta G_{{3}} = G\left( {*{\text{OOH}}} \right){-}G\left( {*{\text{O}}} \right){-}\mu {\text{OH}} = E\left( {*{\text{OOH}}} \right){-}E\left( {*{\text{O}}} \right){-}E\left( {{\text{H}}_{{2}} {\text{O}}} \right) + {1}/{2}E\left( {{\text{H}}_{{2}} } \right){-}eU + \Delta G_{{\text{H}}} + \left( {{\text{pH}}} \right) + \Delta \left( {{\text{ZPE}}{-}T\Delta S} \right)$$

$$\Delta G_{{{4},{5}}} = {4}\cdot\left[ {{1}.{23}\;{\text{eV}}{-}eU + \Delta G_{{\text{H}}} + \left( {{\text{pH}}} \right)} \right]{-}\left( {\Delta G_{{1}} + \Delta G_{{2}} + \Delta G_{{3}} } \right)$$

Herein, *U* represents the potential against the normal hydrogen electrode (NHE) in standard conditions. When pH ≠ 0, ∆*G*_H_ + (pH) is defined as –kBT log (pH), where kB means Boltzman constant, and the kBT = 0.025692 eV (*T* = 298.15 K). ∆*G*_*i*_ is obtained from DFT energy (*E*), zero-point energy (ZPE), and entropy correction. For the *OH, *O and *OOH, the corresponding correction values of ∆(ZPE – TS) are 0.35, 0.05 and 0.4 eV, respectively. In order to exclude the possibility of the energy calculation involving O_2_ (gas) which is hardly obtained within the GGA-DFT scheme, we fixed the sum of ∆*G*_1–5_ as 4.92 eV. The theoretical *η* was accordingly calculated from the ∆G_i_ through the equation: *η* = max [∆*G*_1_, ∆*G*_2_, ∆*G*_3_, ∆*G*_4,5_]/*e* – 1.23 V.

## Results and Discussion

### Synthesis of Bimetallic Oxysulfide Electrocatalysts

**Fig. 1:**
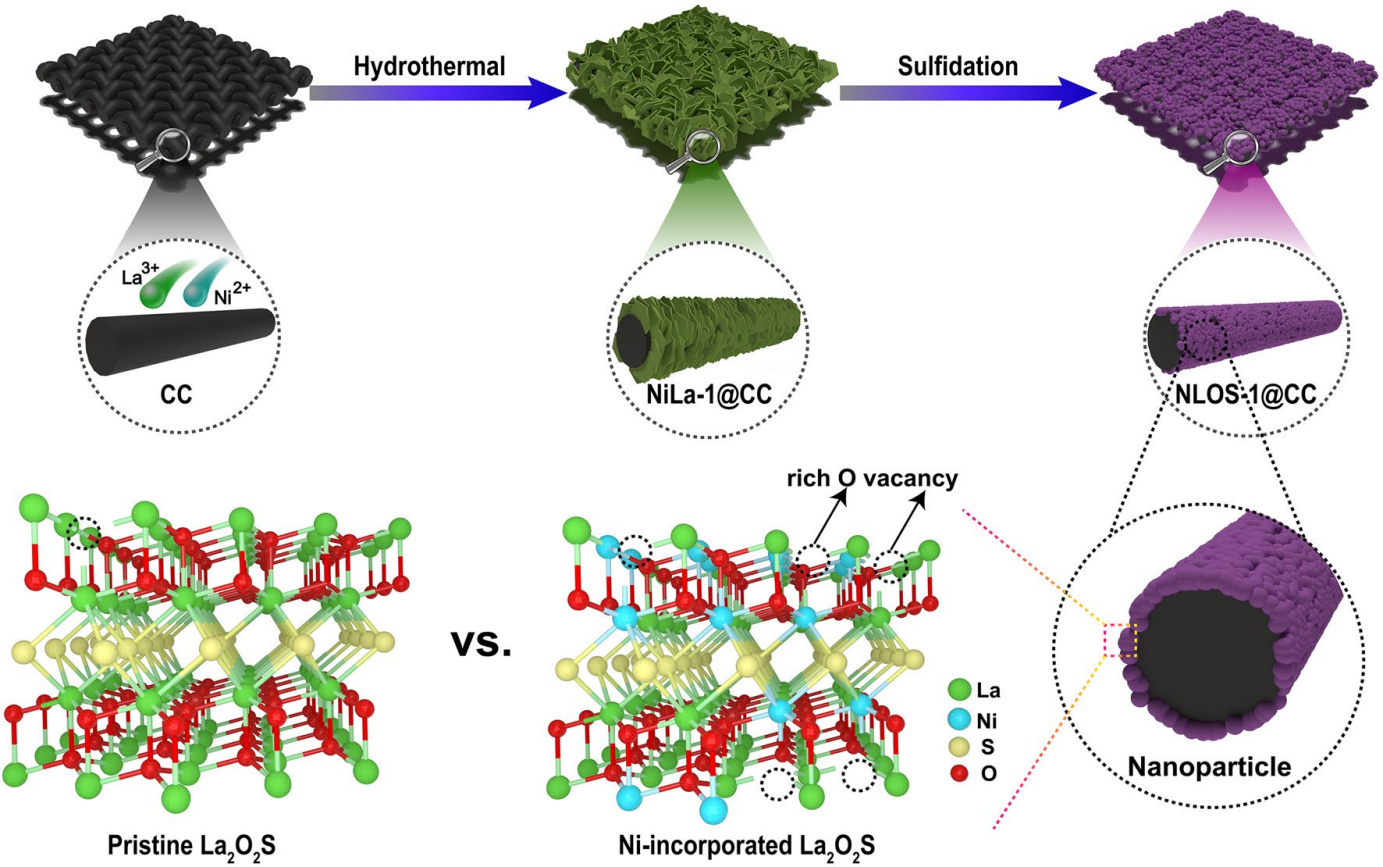
Schematic illustration for the synthesis of NLOS-1@CC

As elaborated in Fig. 1 and the Experimental Section, the optimal electrocatalysts were synthesized through a facile two-step method. The high-crystalline Ni/La hydroxides nanosheet arrays on the carbon cloth surface (NiLa-1@CC) was firstly prepared as precursor using a hydrothermal treatment during which the Ni^2+^ and La^3+^ sources were employed with a molar ratio of 1:1, while hexamethylenetetramine served as the hydrolysis agent [56, 57]. Following that, a sulfidation treatment was performed to convert the precursor nanosheets into the (Ni,La)_2_O_2_S nanoparticles with rich oxygen vacancy anchored on CC (NLOS-1@CC). Other control samples with a molar ratio of Ni to La for 0:1 (NLOS-0@CC), 0.25:1 (NLOS-0.25@CC), 0.5:1 (NLOS-0.5@CC), 2:1 (NLOS-2@CC), and 1:0 (Ni_3_S_2_@CC) were also prepared by the similar procedure.

### Characterizations of Bimetallic Oxysulfide Electrocatalysts

**Fig. 2:**
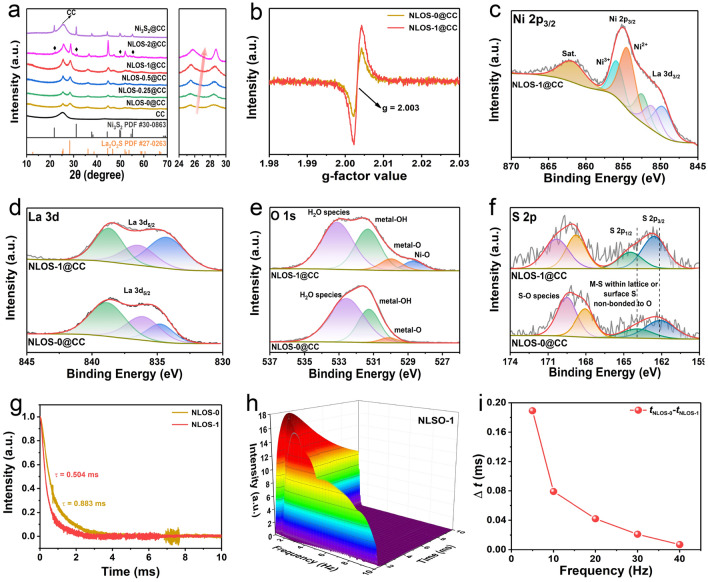
**a** XRD patterns of the as-prepared NLOS-X@CC (X means the ratio of Ni to La: X = 0, 0.25, 0.5, 1, and 2, respectively) and Ni_3_S_2_@CC (left) with the associated magnified part (right) of XRD patterns of NLOS-X@CC. **b** EPR spectra of NLOS-0 and NLOS-1; High-resolution XPS spectrum of **c** Ni 2p_3/2_ XPS for NLOS-1@CC, as well as XPS spectra of **d** La 3d, **e** O 1s, and **f** S 2p for NLOS-0@CC and NLOS-1@CC. **g** TPV curves and corresponding decay times of NLOS-0 and NLOS-1. **h** 3D CWT spectrum of NLOS-1. **i** Time difference (Δ*t*) between NLOS-0 and NLOS-1 in the time-intensity curve (frequencies were fixed at 8, 10, 20, 30, and 40 Hz, respectively)

In order to confirm the phase transformation, powder XRD was employed to detect the products at various stages in the preparation process. The initial XRD patterns identified the formation of Ni_*x*_La_1−*x*_(OH)_3_ precursor phases (PDF #13-0084) (Fig. S1), which were then successfully transformed to Ni-incorporated La_2_O_2_S after sulfidation. Figure 2a displays the XRD pattern of the NLOS-1@CC that showed diffraction peaks corresponding to the La_2_O_2_S-type phase (PDF #27-0263) with additional broad carbon peaks from the CC. The ICP-OES findings confirmed the molar ratio of La to Ni as 1:1.01 (Table S1), which was consistent with the designed precursor. Besides, the XRD patterns of other control samples are also listed and compared in Fig. 2a, from which it could be seen that as the ratio of Ni to La increased from 0:1 to 1:1, the diffraction peaks of (Ni,La)_2_O_2_S gradually shifted to the higher angle, which suggested that the substitution of Ni with a small atomic radius for La induced the lattice contraction. It was worth noting that when the ratio of Ni to La was further increased to 2:1, in addition to the (Ni,La)_2_O_2_S phase, the Ni_3_S_2_ phase also appeared, implying that the saturation solid solubility of Ni in La_2_O_2_S was reached [58], and the excess Ni converted into Ni_3_S_2_ during sulfidation. For the Ni-containing precursor without La, only pristine Ni_3_S_2_ was achieved by sulfidation. The ICP-OES results showed that the proportion of Ni in the product also gradually increased, which was consistent with the expected ones (Table S1). To further understand the effect of Ni incorporation on the phase structure, the EPR spectra of NLOS-0@CC and NLOS-1@CC were measured and compared. As presented in Fig. 2b, the signal for oxygen vacancy was enhanced upon the introduction of Ni into the host lattice of La_2_O_2_S. The XPS was further performed to examine the surface state of elements in NLOS-1@CC, together with NLOS-0@CC for comparison. As shown in Fig. 2c, the high-resolution Ni 2p_3/2_ XPS spectrum of NLOS-1@CC was well deconvoluted into two peaks at 854.7 and 856.0 eV associated with the Ni^2+^ and Ni^3+^, respectively [59]. Because of the partial signal overlap between Ni 2p_3/2_ and La 3d_3/2,_ three small peaks representing La^3+^ (La 3d_3/2_) could also be observed in the high-resolution Ni 2p_3/2_ XPS spectrum [60, 61]. Correspondingly, the three peaks assigned to the La^3+^ were marked within around 828–842 eV of the high-resolution La 3d_5/2_ XPS spectra for NLOS-1@CC, which were basically unchanged as compared to those for NLOS-0@CC (Fig. 2d) [41, 62]. Besides, both high-resolution O 1s XPS spectra in NLOS-0 and NLOS-1 were consistent with that of La_2_O_2_S reported previously (Fig. 2e) [62]. Remarkably, an additional peak typical for the Ni–O bond could be identified at around 528.6 eV in NLOS-1, further demonstrating the successful incorporation of Ni species into La_2_O_2_S [63]. According to the previous report on La_2_O_2_S [64], the fitted peaks in the high-resolution S 2p XPS spectrum of NLOS-0@CC might be assigned to the presence of M-S within lattice or surface S non-bonded to O, as well as S–O species [65], while these peaks slightly shifted to higher binding energy in NLOS-1@CC, suggesting the electron donation from S atoms after the introduction of Ni atoms (Fig. 2f). Such enhancement of oxygen vacancies and peaks shift of sulfur could be due to the fact that the partial substitution of high valence La species with a lower oxidation state of Ni species leads to the charge redistribution of the element to maintain the charge neutrality of the system [19]. Given the above findings, the TPV technique was carried out to probe how the incorporation of Ni influences the interfacial charge transfer kinetics of La_2_O_2_S during electrocatalysis (Fig. S2) [66–69]. As shown in Fig. 2g, the time decay constant (*τ*) values were extracted from TPV curves to reflect the decay rate of NLOS-0 and NLOS-1. The lower *τ* value means a faster interfacial charge transfer [70]. It could be found that the *τ* value of NLOS-1 (0.504 ms) was distinctly smaller than that of NLOS-0 (0.883 ms), indicating the accelerated interfacial charge transfer upon the introduction of Ni. Notably, considering that the interfacial transport processes with different speeds existed during the whole decay process, Fast Fourier Transform (FFT) patterns were also acquired from TPV data [71]. As shown in Fig. S3, FFT curves of both NLOS-0 and NLOS-1 displayed a series of continuous signals with invisible peaks, substantiating that no evident static or periodic frequency components emerged in the TPV relaxation signals. Besides, we also employed the three-dimensional (3D) Continuous Wavelet Transform (CWT) to explore various decay processes for NLOS-0 and NLOS-1, which could reflect the correlation among time, frequency, and intensity factors (Figs. 2h and S4a). Based on the CWT, we calculated the time difference (Δ*t* = *t*_1_–*t*_2_, *t*_1_ and *t*_2_ corresponds to the peak time for NLOS-0 and NLOS-1, respectively) between NLOS-0 and NLOS-1 at a wide range frequency from 8 to 40 Hz. As demonstrated in Fig. S4b–f, the *t*_2_ values within the entire frequency region were always smaller than *t*_1_ values, revealing that NLOS-1 with Ni incorporation facilitates a more rapid transport of interfacial electrons compared to the pristine NLOS-0 through the whole process. However, with the increment of frequency, the time difference (Δ*t* = *t*_1_–*t*_2_) was gradually decreased, which almost dropped to zero at the highest frequency of 40 Hz (Fig. 2i). Such a variation tendency demonstrates that the most pronounced difference for transfer kinetics of interfacial electrons mainly occurred at low frequency regions. Overall, the incorporation of Ni atoms enabled La_2_O_2_S to accelerate electron transfer at the interface, which was expected to promote the oxidation reaction of LaNiO_2_S electrocatalyst in an alkaline electrolyte (OH^–^ ions) [72, 73].

**Fig. 3:**
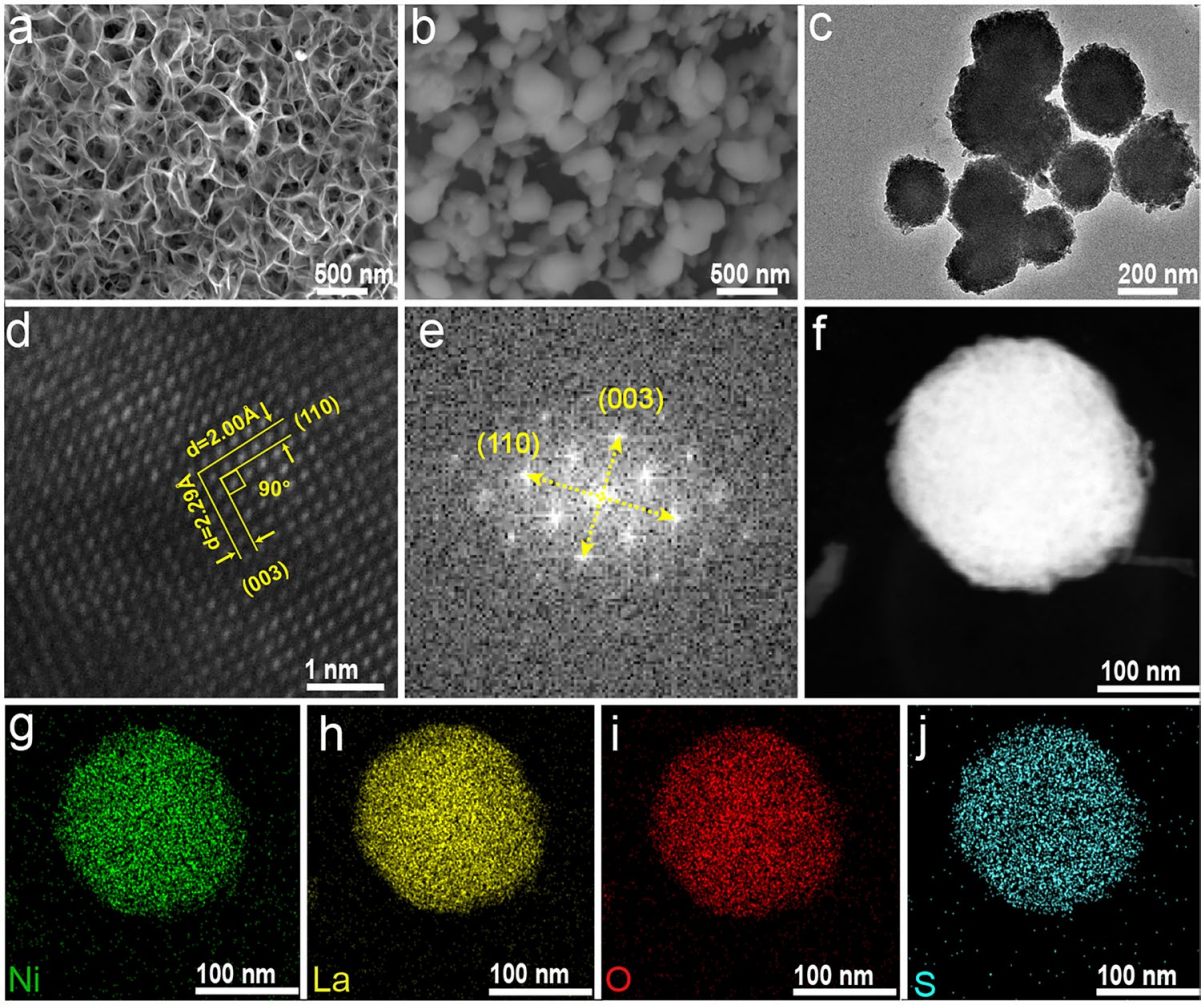
FESEM images of **a** NiLa-1 precursor and **b** the resulting NLOS-1 nanoparticles. **c** TEM and **d** its HRTEM images, as well as the associated **e** FFT pattern of NLOS-1. **f** HAADF-STEM pattern of NLOS-1 nanoparticle and its corresponding EDS mapping images of **g** Ni, **h** La, **i** O, and** j** S

On the other hand, the morphology evolution during the synthetic process as well as the effect of Ni incorporation on morphology was examined by FESEM. In terms of the precursors without Ni incorporating, a large number of La(OH)_3_ particles were densely covered onto the surface of CC (Fig. S5a). However, upon the presence of Ni, the spherical particles changed into the nanosheet arrays, which became more apparent with the increase in Ni content (Figs. 3a and S5b–f). After sulfidation, the pristine La_2_O_2_S with particle sizes around micrometer transformed from La(OH)_3_ aggregated severely (Fig. S6a). Interestingly, those nanosheet precursors containing Ni species also transformed to (Ni, La)_2_O_2_S nanoparticles due to the thermal conversion and new phase crystallization. Nonetheless, after a careful examination, it could be found that those (Ni_,_ La)_2_O_2_S presented much smaller particle size and more uniform distribution onto the surface of CC (Figs. 3b and S6b–f), which was probably due to the certain space distance between nanosheets in precursors that prevented the formed particles from agglomeration to a large extent. In order to further decouple the microstructure (Ni_,_ La)_2_O_2_S, NLOS-1 was selected as the representative example to perform TEM characterization. As shown in Fig. 3c, particles with an average size of 200 nm were clearly observed. Further, high-resolution TEM (HRTEM) image depicted well-discernible lattice fringes with distances of 2.00 and 2.29 Å (Fig. 3d), which corresponded to the (110) and (003) facets of La_2_O_2_S-type phase with an intersection angle of 90°, respectively [74, 75]. Note that these two lattice fringe distances were slightly smaller than those of the reported standard La_2_O_2_S phase, which has the theoretic values of 2.02 and 2.31 Å for (110) and (003) facets, respectively (PDF #27-0263), demonstrating again the lattice contraction of La_2_O_2_S by substitution of La with Ni. The presence of such two crystal planes of La_2_O_2_S can be further proven by the associated FFT patterns (Fig. 3e). These observations also illustrated that NLOS-1 well inherited the host lattice structure of La_2_O_2_S, being in accordance with the XRD findings. Moreover, its high-angle annular dark-field scanning transmission electron microscopy (HAADF-STEM) pattern and corresponding EDS elemental mapping images displayed the uniform distribution of Ni, La, O, and S elements within the NLOS-1 nanoparticle, confirming that Ni atoms were evenly incorporated into La_2_O_2_S host (Fig. 3f–j).

### Electrocatalytic OER Performances

**Fig. 4:**
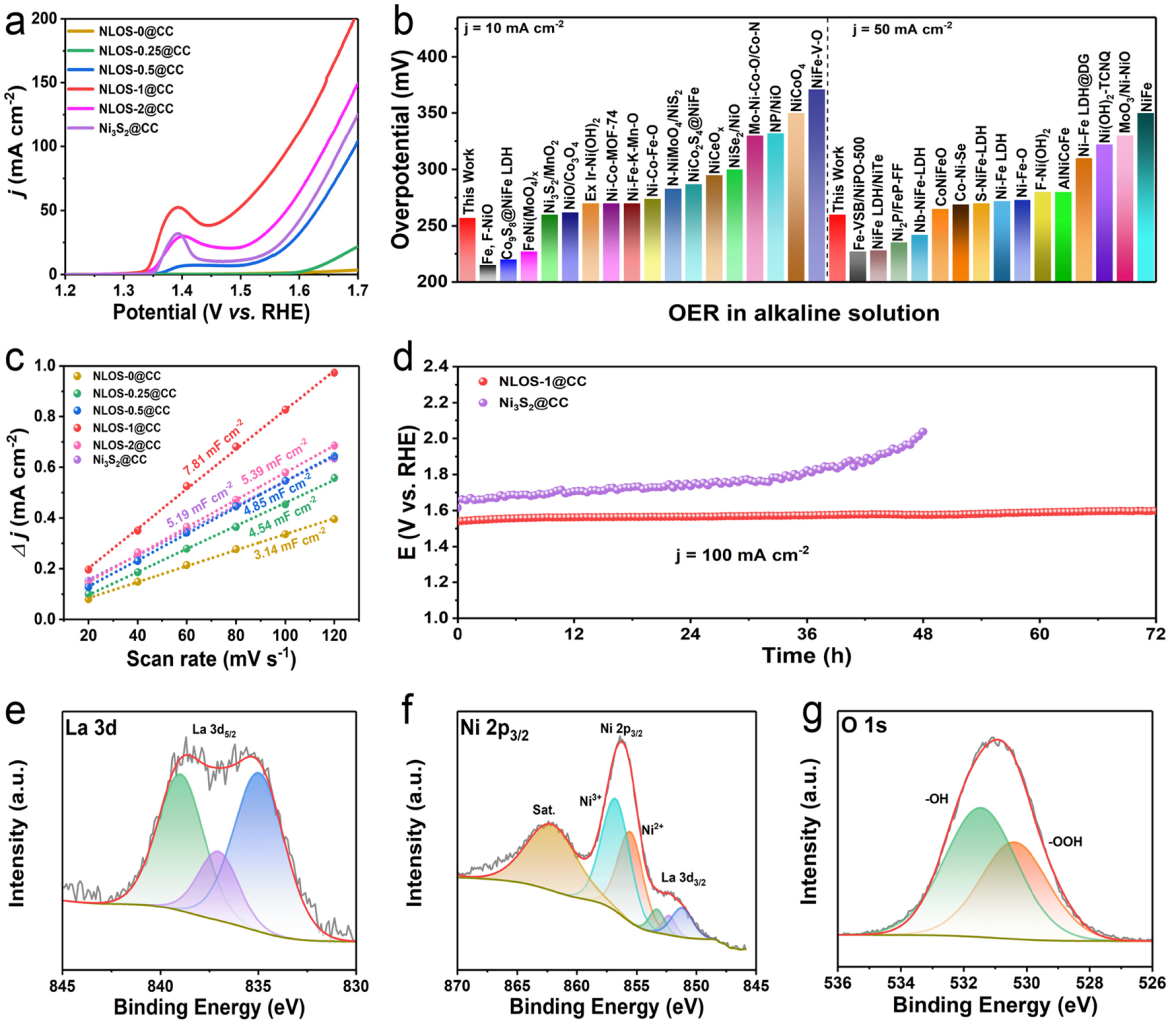
**a** IR-corrected LSV polarization curves. **b** The comparison of alkaline OER activity of NLOS-1@CC with other recently reported Ni-based OER electrocatalysts. **c** *C*_dl_ of NLOS-X@CC (X = 0, 0.25, 0.5, 1, and 2, respectively) and Ni_3_S_2_@CC. **d** Long-term OER CP tests of NLOS-1@CC and Ni_3_S_2_@CC under a constant current density of 100 mA cm^–2^. High-resolution XPS spectra of post-OER NLOS-1@CC: **e** La 3d, **f** Ni 2p, and **g** O1s

As the well-defined NLOS-1@CC electrode that meets our expectations was achieved, we then evaluated its OER performance in 1 M KOH by using a standard three-electrode system. Before performing electrochemistry, the reference electrode (Hg/HgO) was calibrated to the RHE in hydrogen-saturated 1 M KOH (Fig. S7). For better illustration, the OER performance of the above-mentioned control electrodes, as well as commercial IrO_2_/CC and CC substrate, were also evaluated under identical conditions. Figures 4a and S8 display the LSV curves, from which NLOS-1@CC required only an overpotential of 260 mV to deliver the current density of 50 mA cm^–2^, which was the lowest among all the tested electrocatalysts. It is worth noting that NLOS-0@CC with the absence of Ni species showed negligible OER activity, implying the poor intrinsic OER activity of La species. Furthermore, from the OER LSV curves recorded in Fig. 4a, we could observe that: (1) almost no oxidation peaks could be found for NLOS-0@CC and NLOS-0.25@CC during the OER LSV test, while the oxidation peaks of other samples all apparently emerged at around 1.37 V, suggesting that the obvious oxidation of Ni^2+^ to Ni^3+^ occurred with the incorporation of sufficient Ni atoms [76]; (2) The oxidation peak areas for the abovementioned catalysts increased in the order of NLOS-0.5@CC, Ni_3_S_2_@CC, NLOS-2@CC, and NLOS-1@CC, in agreement with their activity trend [76]. Meanwhile, NLOS-0@CC and NLOS-0.25@CC without oxidation peaks displayed almost no OER activity; (3) The above results implied that the higher-valence Ni atoms were the real active sites for water oxidation. Besides, although increasing the incorporated Ni atoms to some extent could positively boost the oxidized higher-valent Ni sites, further incorporation would result in the phase change or morphology aggregation, thus leading to the unavailability of the active Ni atoms and degrading the OER activity. Therefore, NLOS-1@CC possessed the optimum Ni content. To rule out the effect of oxidation peak on the evaluation of OER activity, the LSV curve NLOS-1@CC was also tested in the negative scan direction (Fig. S9). As expected, from which the overpotential at 10 mA cm^–2^ was as low as 257 mV, further confirming the outstanding OER activity of NLOS-1@CC, which outmatched most of the previously documented Ni-based alkaline OER catalysts (Fig. 4b and Table S2). In view of the fact that CC substrate cannot be stabilized at high current density, we specially deposited NLOS-1 on NF substrate, which can drive industrially related current density of 500 mA cm^−2^ (Fig. S10), implying its promising potential for practical application. To uncover the origin for the superior OER activity, the Tafel slopes of NLOS-1@CC and control samples were calculated and the results showed that NLOS-1@CC exhibited the most favorable OER kinetics with the lowest value of Tafel slope (88.2 mV dec^−1^) (Fig. S11). The EIS measurements were also carried out to uncover the charge-transfer resistance (*R*_ct_) from the fitted Nyquist plots (Fig. S12 and Table S3), where NLOS-1@CC exhibited the smallest *R*_ct_ value (11.1 Ω) among all the probed electrodes, again manifesting its exceptional charge transfer kinetics. Moreover, because the ECSA is another important factor to influence the OER performance of the catalysts, *C*_dl_ which was linearly proportional to ECSA was determined for NLOS-X@CC (X = 0, 0.25, 0.5, 1, and 2) and Ni_3_S_2_@CC via performing their CV curves within a non-Faradic region (Fig. S13). As shown in Fig. 4c, the *C*_dl_ of NLOS-1@CC (7.81 mF cm^−2^) was much higher than those of NLOS-2@CC (5.39 mF cm^−2^), Ni_3_S_2_@CC (5.19 mF cm^−2^), NLOS-0.5@CC (4.85 mF cm^−2^), NLOS-0.25@CC (4.54 mF cm^−2^), and NLOS-0@CC (3.14 mF cm^−2^), indicating the highly accessible potential active sites in NLOS-1@CC. To better reflect the excellent intrinsic catalytic activity of NLOS-1@CC, the corresponding *C*_dl_-normalized and mass-normalized LSV curves were further attained and shown in Fig. S14 [26, 29], from which NLOS-1@CC also showed the lowest overpotential among all the tested electrodes. Motivated by the exceptional OER activity of NLOS-1@CC, The CP measurement for NLOS-1@CC was operated at a constant current density of 100 mA cm^−2^ to examine its long-term stability. Notably, the applied potential (1.54 V vs. RHE) was unchanged over 72 h, suggesting its robust durability in alkaline media (Fig. 4d). In contrast, the Ni_3_S_2_@CC without any La species required a much higher potential under the same applied current density, and the applied potential continuously increased within 48 h, suggesting the important role of La species in suppressing the leaching of active Ni atoms and thus stabilizing the OER activity (Fig. 4d and Table S5).

### Ex Situ/In Situ Post-OER Characterizations and Theoretical Calculations

To clarify the reasons for the exceptional stability, a series of ex situ and in situ characterizations were adopted for post-OER NLOS-1@CC. As shown in the XPS spectra of Fig. 4e, the La species in the post-OER NLOS-1@CC were still preserved in the form of La^3+^. Compared to this, peaks assigned to Ni species shifted to higher binding energy after the OER stability tests (Fig. 4f). Moreover, the concentration ratio of Ni^3+^/Ni^2+^ (1.69) within the Ni 2p_3/2_ XPS spectra of the post-OER NLOS-1@CC was apparently higher than that of the one before OER (0.70), meaning the formation of Ni-based (oxy)hydroxides with higher oxidation state [77, 78]. Interestingly, the oxyanion (SO_4_^2−^) concurrently emerged, as identified in S 2p XPS (Fig. S15), meaning the surface adsorption of SO_4_^2−^ during the reconstruction of the La_2_O_2_S host [13, 30]. Furthermore, two peaks positioned at around 530.4 and 531.4 eV in the high-resolution O 1s XPS spectrum of post-OER NLOS-1@CC could be correlated with the hydroxylation of the catalyst (Fig. 4g) [18]. The above observations strongly indicated the occurrence of OER-induced phase reconstruction. This point was further validated by the increase in *C*_dl_ value (Fig. S16) and the variation of the XRD pattern for NLOS-1@CC after the OER tests (Fig. S17).

**Fig. 5:**
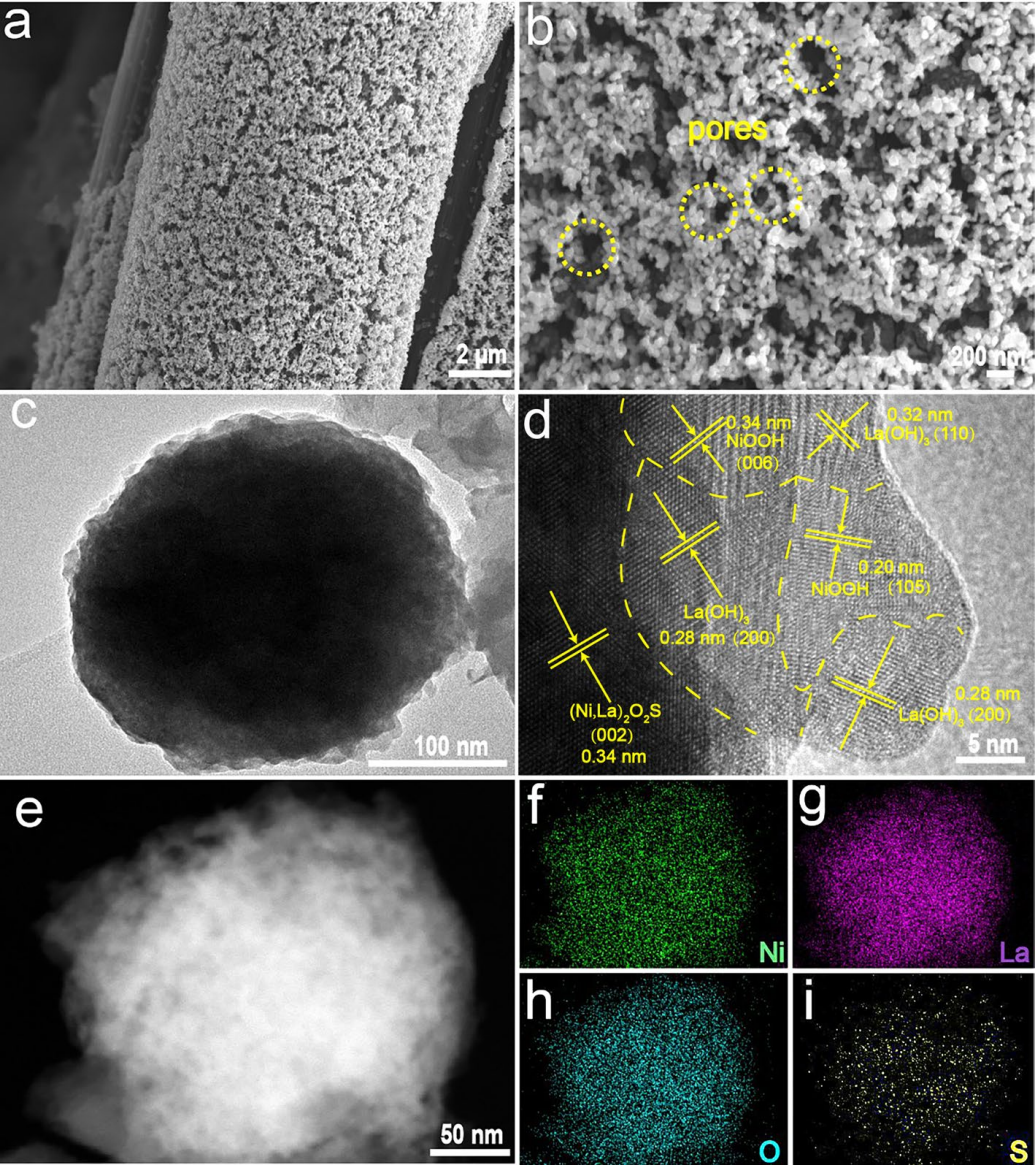
**a**, **b** FESEM images of post-OER NLOS-1@CC; **c** TEM image, **d** HRTEM image, and **e** HAADF-STEM pattern of post-OER NLOS-1 nanoparticle and its corresponding EDS mapping images of **f** Ni, **g** La, **h** O, and **i** S

The morphology and microstructure of post-OER NLOS-1 was examined by FESEM and TEM. As shown in FESEM images of post-OER NLOS-1@CC, although the nanoparticle morphology was well maintained, it presented a more porous feature between particles (Fig. 5a–b). The TEM image recorded from post-OER NLOS-1 nanoparticle showed a homogenous distribution of numerous nanocrystals (Figs. 5c and S18). The HRTEM image further unveiled that apart from residual (Ni, La)_2_O_2_S (PDF # 27-0263), the post-OER particle was composed of La(OH)_3_ (PDF #13–0084) and NiOOH (PDF #6-75) nanocrystals (the depth of reconstruction region was around 20–30 nm) (Fig. 5d), affirming the surface phase transformation during the OER process. Moreover, the newly formed NiOOH nanocrystals were evenly separated by La(OH)_3_ nanocrystals, which suppressed the severe agglomeration of in situ formed catalytic active NiOOH species, ensuring the sufficient exposure of active surface sites. Furthermore, the HAADF-STEM pattern of post-OER NLOS-1 nanoparticle is also shown in Fig. 5e, from which a highly porous surface could be well identified, contributing to the mass diffusion during catalysis [79, 80]. The corresponding EDS elemental mapping images demonstrated the homogenous distribution of conspicuous La, Ni, and O, while much sparser S could be observed (Fig. 5f–i), implying the deep reconstruction of NLOS into NiOOH/La(OH)_3_ along with leaching of S atoms during OER. Furthermore, according to the corresponding EDS data of NLOS-1 before and after OER CP, no significant impurity Fe atoms were doped into the reconstructed species (Table S4), indicating the activity was mainly contributed by the Ni species.

**Fig. 6:**
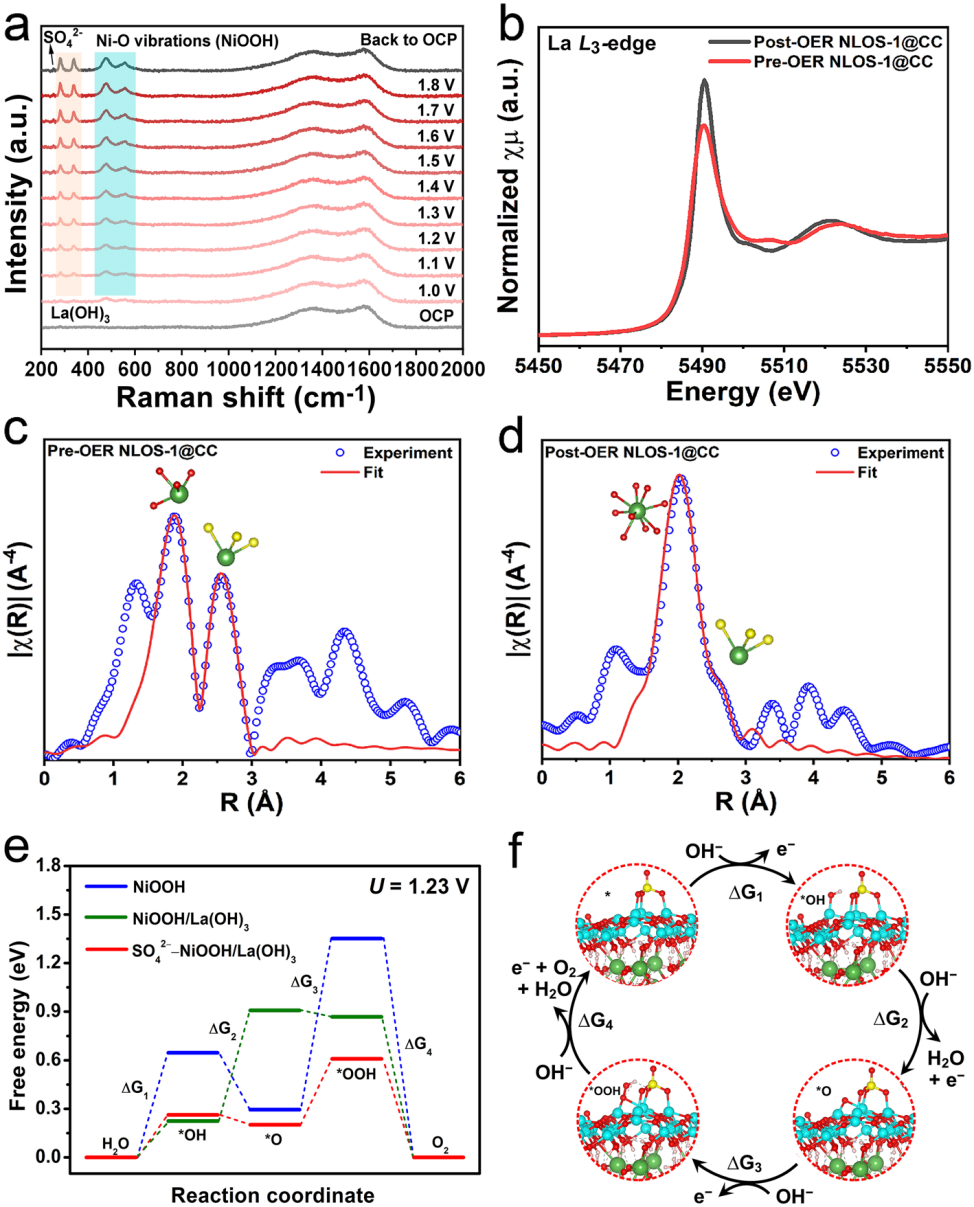
**a** Raman spectra at OCP, before and after electrochemistry, as well as the in situ Raman results of NLOS-1@CC under the applied potential from 1.0 to 1.8 V (vs. RHE) with an increasing interval of 0.1 V. **b** La *L*_3_-edge XANES spectra of NLOS-1 before and after OER CP. La *L*_3_-edge Fourier transformed (FT)-EXAFS spectra of NLOS-1 **c** before and **d** after OER CP fitted in *R* space. **e** Free energy diagrams of alkaline water oxidation at 1.23 V (vs. RHE) for NiOOH, NiOOH/La(OH)_3_, and SO_4_^2−^−NiOOH/La(OH)_3_. **f** Alkaline OER routes based on the active site (Ni) of SO_4_^2−^−NiOOH/La(OH)_3_. Atoms with cyan, green, red, yellow, and pink colors represent Ni, La, O, S, and H atoms, respectively

The ex and in situ Raman, pre- and post-OER X-ray absorption (XAS), as well as DFT, were further conducted to acquire insights into the phase transformation, as well as the role of La in immobilizing oxyanion (SO_4_^2−^) and promoting OER performance. First, an assembled Raman-electrochemistry system was applied in situ to track the OER-driven phase evolution of Ni-modified La_2_S_2_O (Fig. 6a). At the OCP, NLOS-1@CC only exhibited two peaks at around 1350 and 1580 cm^−1^, which could be attributed to the D-band and G-band of carbon from the CC substrate [81]. Upon initialing OER, two new peaks emerged at around 475 and 557 cm^−1^, which corresponded to the bending and stretching of the Ni–O bond in NiOOH, respectively [82, 83]. Meanwhile, two other small new peaks at 280 and 339 cm^−1^ were responsible for characteristic signals of the OH^–^ stretching model and La translation mode of La(OH)_3_, respectively [84]. With the continuous increase in the applied anodic potential, all of these four peaks gradually intensified, which could still be well-retained even when the applied potential was reverted to OCP. It is worth pointing out that such a trend was also recorded for the band at around 252 cm^−1^, which represents the existence of SO_4_^2−^ [85]. Besides, the time-dependent in situ Raman on NLOS-1@CC was also carried out, which was fixed at 1.7 V vs. RHE where rigorous OER took place. As was depicted in Fig. S19, the emerging of bands assigned to NiOOH, La(OH)_3_, and SO_4_^2−^ from NLOS-1 was initiated at around 10 min, and became intensified until 60 min. Afterward, those bands appeared to be stabilized, which signified that the surface reconstruction was probably started at only around 10 min and completed at around 60 min. These phenomena directly elucidated that a rapid phase conversion was triggered during the anodic alkaline OER process, i.e., surface La and Ni species for NLOS-1 were swiftly converted into La(OH)_3_ and NiOOH, respectively, both of which exhibited excellent phase stability even in the case the applied potential returned to OCP. Simultaneously, the surface S of NLOS-1 was also rapidly oxidized. Apart from dissolving into the electrolyte instead of being re-deposited on the cathode, some leached S atoms form the surface of pristine NLOS transformed into SO_4_^2−^, steadily adsorbed on the surface of the reconstructed heterostructure. This claim can be further confirmed through the XPS, Raman, and EDS (atomic ratio) findings of the NLOS-1@CC sample, EDS results of the graphite rod (serving as the counter electrode during the tests, Fig. S20), as well as the ICP-OES results of the associated electrolyte after long-term OER CP (Figs. S15 and S21, Tables S4 and S5). Since the transformation of Ni into NiOOH during alkaline OER has commonly been observed in previous reports, herein, the XAS spectra of La *L*_3_-edge in NLOS-1 before and after OER CP was measured and compared to confirm the variation of chemical state and local structure of La species. As revealed by the La *L*_3_-edge X-ray absorption near-edge structure (XANES) results in Fig. 6b, the white-line peak of post-OER NLOS-1 emerged at almost the same intensity compared as that of pre-OER NLOS-1, indicating that La atoms in Ni-incorporated La_2_O_2_S well maintained their oxidation state (+ 3) during OER process, which was indeed in accordance with the XPS results. However, by comparing the La *L*-edge extended X-ray absorption fine structure (EXAFS) profiles of pre- and post-OER NLOS-1, it could be found that pre-OER NLOS-1 presented two prominent peaks at around 1.87 and 2.55 Å, corresponding to La–O and La-S coordination structure of NiLaO_2_S phase, respectively (Fig. 6c and Table S6). Remarkably, after OER CP, the peak assigned to the presence of La–O positively shifted to around 2.06 Å accompanied by the minor shoulder peak representing La-S coordination at around 2.67 Å (Fig. 6d and Table S6), meaning the dominant La(OH)_3_ phase accompanied with small amount of residual NiLaO_2_S. Such variations corroborated the OER-induced surface structural evolution of La(OH)_3_ from the NiLaO_2_S host. Combined with the observations on the emerged SO_4_^2−^ adsorbates on the reconstructed heterostructure, DFT calculations were performed to compare the adsorption energy of SO_4_^2−^ (E_ad-sulfate_) on the surface of NiOOH and NiOOH/La(OH)_3_ models (see their crystal structure models from the top-and cross-sectional views in Fig. S22). As seen in Fig. S22, the *E*_ad-sulfate_ for NiOOH/La(OH)_3_ was −3.78 eV, which was much more negative than that (− 0.56 eV) for pristine NiOOH. This strongly demonstrated that the presence of La(OH)_3_ could greatly improve the adsorption ability of NiOOH to SO_4_^2−^, while pristine NiOOH indeed showed a weak ability to capture SO_4_^2−^. Figure 6e further depicted the Gibbs free energy diagrams of OER intermediates over NiOOH, NiOOH/La(OH)_3_, and SO_4_^2−^-NiOOH/La(OH)_3_ models. Noticeably, according to Fig. 6e, it could be known that the overpotential for SO_4_^2−^-NiOOH/La(OH)_3_ was 0.410 V, which is much smaller than that for NiOOH (1.006 V) and NiOOH/La(OH)_3_ (0.699 V). This result confirmed the adsorption of SO_4_^2−^ could strikingly modulate the electronic structure of NiOOH/La(OH)_3_ and optimized the Gibbs free energy of OER intermediates, facilitating the OER reaction (Fig. 6f). The above research results also inspire us, that is, by reconstructing appropriate heterojunction, the oxyanions can be effectively adsorbed and stabilized, and thus an improvement in OER activity and durability.

## Conclusions

In summary, we have reported an unprecedented nickel-lanthanum oxysulfide (NLOS) that was derived from the La_2_O_2_S prototype as a binder-free precatalyst for highly active and ultrastable alkaline OER. The synergistic effect among Ni, La, S, and O during OER contributed to the deep reconstruction of the NLOS surface into a porous heterostructure consisting of the in situ formed NiOOH nanodomains strongly coupled with La(OH)_3_ nanofences. Furthermore, the presence of La(OH)_3_ enabled NiOOH to easily adsorb and stabilize the SO_4_^2−^ anions during OER. Such a unique heterostructure assured the active sites exposure, charge transfer acceleration, robust structural stability as well as the diminishing of the adsorption free energy barrier toward OER. Benefiting from these merits, the optimized NLOS@CC electrocatalyst only required an ultralow overpotential of 260 mV at 50 mA cm^−2^ and delivered impressive durability over 3 days at 100 mA cm^−2^, outperforming most reported Ni-based alkaline OER catalysts. The materials developed in this contribution may inspire a series of novel rare earth metal/transition metal-based electrocatalysts and the mechanistic understanding of La in promoting OER activity as well as may also provide new directions into the design of well-defined precatalysts.

### Supplementary Information

Below is the link to the electronic supplementary material.

Supplementary DOC (DOCX 3758 kb)
