# Supplementary material for: Immobilization of Oxyanions on the Reconstructed Heterostructure Evolved from a Bimetallic Oxysulfide for the Promotion of Oxygen Evolution Reaction
Source: Nanomicro Lett. 2023 Jul 29;15:186. doi: 10.1007/s40820-023-01164-9 (PMC10387036; doi:10.1007/s40820-023-01164-9)
Supplement: Supplementary file 1 — Supplementary DOC (DOCX 3758 kb) [file 40820_2023_1164_MOESM1_ESM.docx]

Supporting Information for

**Immobilization of oxyanions on the reconstructed heterostructure evolved from a bimetallic oxysulfide for the promotion of oxygen evolution reaction**

Kai Yu^1^, Hongyuan Yang^2^, Hao Zhang^1^, Hui Huang^1^, Zhaowu Wang^4^, Zhenhui Kang^1,^ *, Yang Liu^1^, Prashanth W. Menezes^2, 3,^ *, and Ziliang Chen^1, 2,^ *

^1^Institute of Functional Nano and Soft Materials (FUNSOM), Jiangsu Key Laboratory for Carbon-based Functional Materials and Devices, Joint International Research Laboratory of Carbon-Based Functional Materials and Devices, Soochow University, Suzhou 215123, P. R. China

^2^Department of Chemistry: Metalorganics and Inorganic Materials, Technical University of Berlin, Straße des 17 Juni 135. Sekr. C2, Berlin 10623, Germany

^3^Materials Chemistry Group for Thin Film Catalysis – CatLab, Helmholtz-Zentrum Berlin für Materialien und Energie, Albert-Einstein-Str. 15, Berlin 12489, Germany

^4^School of Physics and Engineering, Longmen Loboratory, Henan University of Science and Technology, Luoyang, 471023, P. R. China

Kai Yu and Hongyuan Yang contributed equally to this work.

*Corresponding author. E-mail: zhkang@suda.edu.cn; prashanth.menezes@mailbox.tu-berlin.de; prashanth.menezes@helmholtz-berlin.de; zlchen@suda.edu.cn

**Supplementary Figures and Tables**





**Fig. S1** XRD patterns of NiLa-X@CC and Ni LDH@CC precursors.

Note that when the molar ratio of the Ni/La source ranged from 0 to 1, the only presented phase of all the samples was La(OH)_3_ (PDF #13-0084), indicating the precursors were Ni*_x_*La_1-_*_x_*(OH)_3_. Moreover, after increasing the ratio of Ni and La to 2 (NiLa-2), the only phase which can be identified was a typical LDH phase (PDF #30-1835) [S1, S2], thus the associated compounds were considered as NiLa LDH. When the Ni content was continuously increased without any addition of La source, a pure Ni LDH phase can be observed in the sample of Ni LDH@CC.


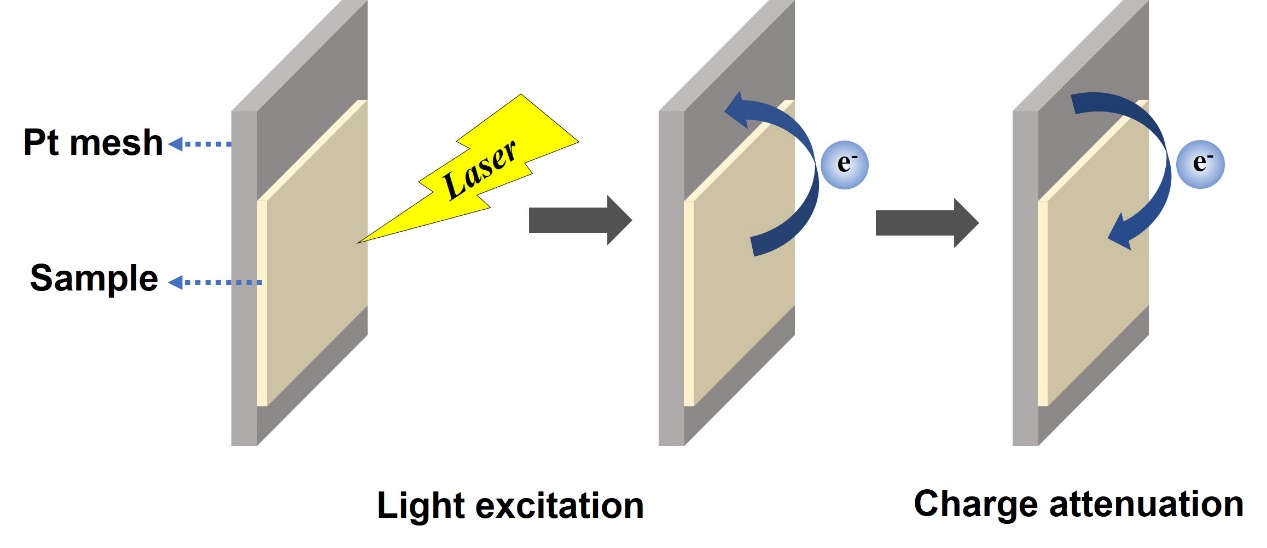


**Fig. S2** Schematic diagram of transient photo-induced voltage setup and its working mechanism.

The TPV test system is used to characterize and analyze the kinetic processes of photoelectron extraction and interfacial charge transport in materials, which is schematically demonstrated in this figure. A laser pulse, whose wavelength is 355 nm and pulse width is 5 ns, generated from the third harmonic Nd: YAG laser instrument is applied to the surface of the powder samples supported on a platinum mesh substrate, inducing the separation and transfer of photogenerated charges within those samples. The resulting potential difference on the sample surface is detected by the connected platinum mesh and oscilloscope. Subsequently, the potential change is captured by the information acquisition card to obtain the corresponding transient photovoltage signal. Herein, the change of photo-induced voltage is recorded at a more precise time span, which can more deeply analyze the properties and mechanism of the material.





**Fig. S3** FFT patterns of NLOS-0 and NLOS-1 obtained from TPV data.


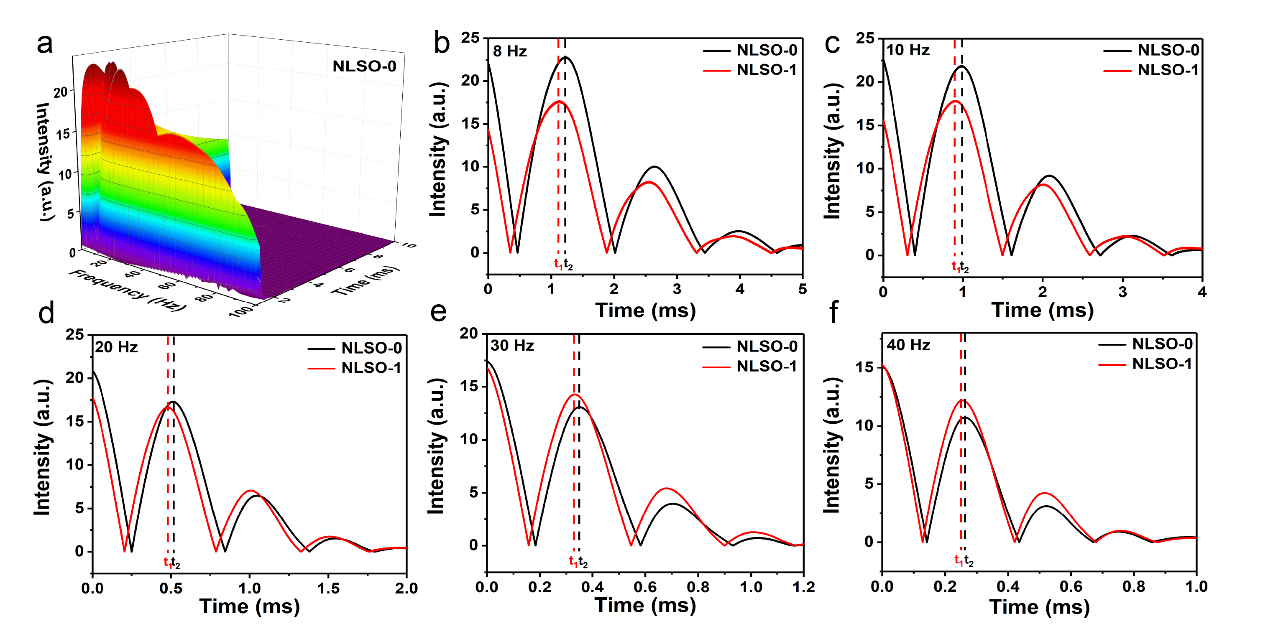


**Fig. S4 a** Three-dimensional CWT spectrum of NLOS-0. Relationships between intensity and time of peak positions at different frequencies: intensity-time curves of NLOS-0 and NLOS-1 (t_1_ and t_2_ are the peak occurrence time of NLOS-0 and NLOS-1, respectively) at the frequency of **b** 8, **c** 10, **d** 20, **e** 30, and **f** 40 Hz.


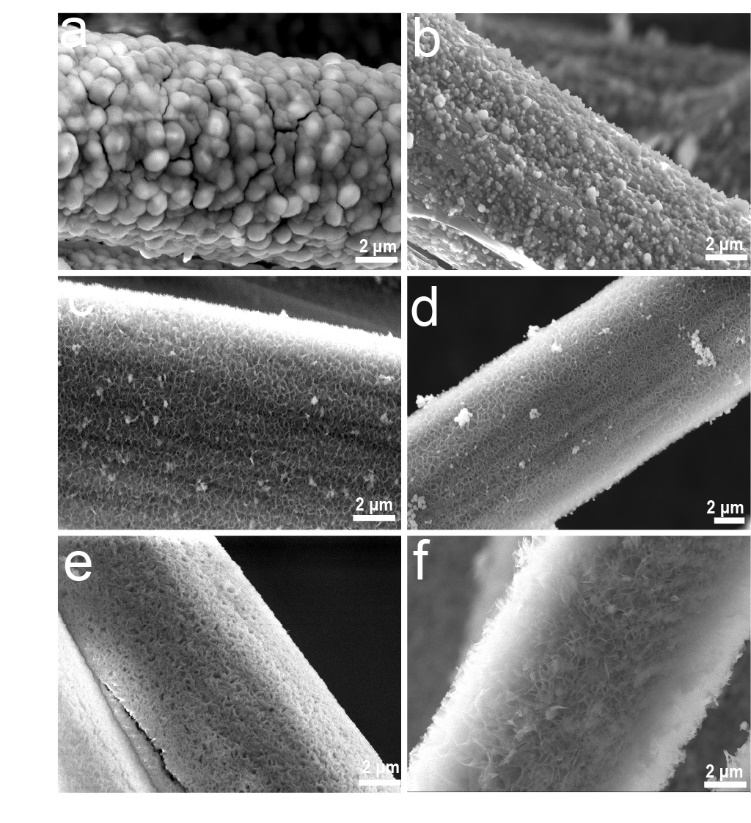


**Fig. S5** FESEM images of **a** NiLa-0@CC, **b** NiLa-0.25@CC, **c** NiLa-0.5@CC, **d** NiLa-1@CC, **e** NiLa-2@CC, and **f** Ni LDH@CC.


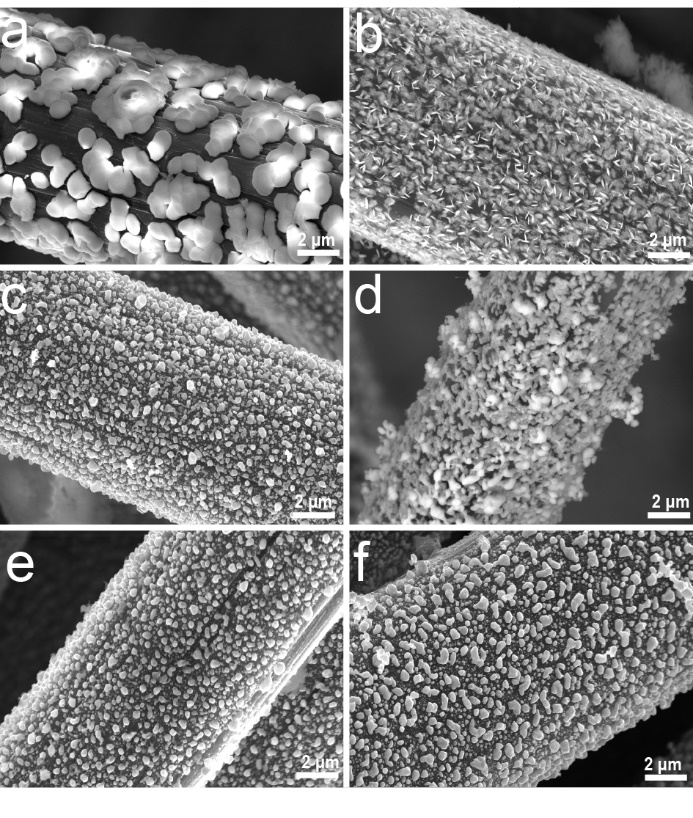


**Fig. S6** FESEM images of **a** NLOS-0@CC, **b** NLOS-0.25@CC, **c** NLOS-0.5@CC, **d** NLOS-1@CC, **e** NLOS-2@CC, and **f** Ni_3_S_2_@CC.

**

**

**Fig. S7** The polarization curves at a scan rate of 1 mV s^-1^ in hydrogen-saturated 1 M KOH (Hg/HgO as the reference electrode).

**

**

**Fig. S8** IR-corrected LSV polarization curves of NiLa-1@CC, CC, NLOS-1@CC and IrO_2_@CC in 1 M KOH electrolyte.





**Fig. S9** IR-corrected LSV polarization curves of NLOS-1 obtained by the negative scan.

**

**

**Fig. S10** IR-corrected LSV polarization curves of NLOS-1@NF.

**

**

**Fig. S11** Tafel plots of NLOS-X@CC (X=0, 0.25, 0.5, 1, and 2, respectively) and Ni_3_S_2_@CC in 1 M KOH electrolyte.


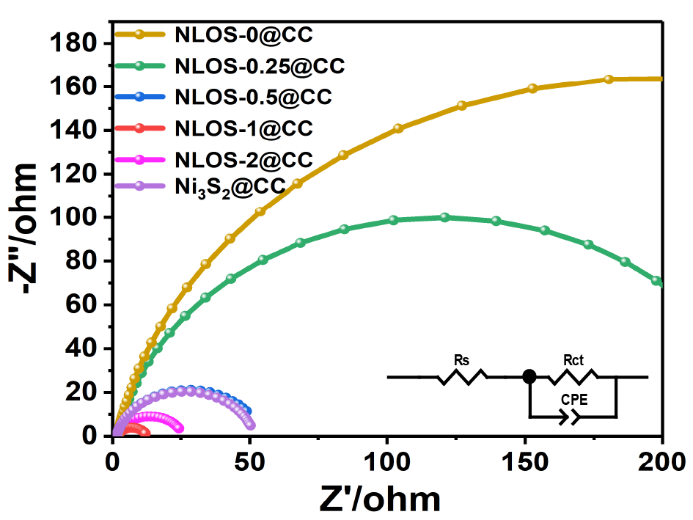


**Fig. S12** Fitted EIS plots of NLOS-X@CC (X=0, 0.25, 0.5, 1, and 2, respectively) and Ni_3_S_2_@CC in 1 M KOH electrolyte at a catalytically active potential of 1.41 V *vs.* RHE in 1 M KOH with an inset of the equivalent circuit.


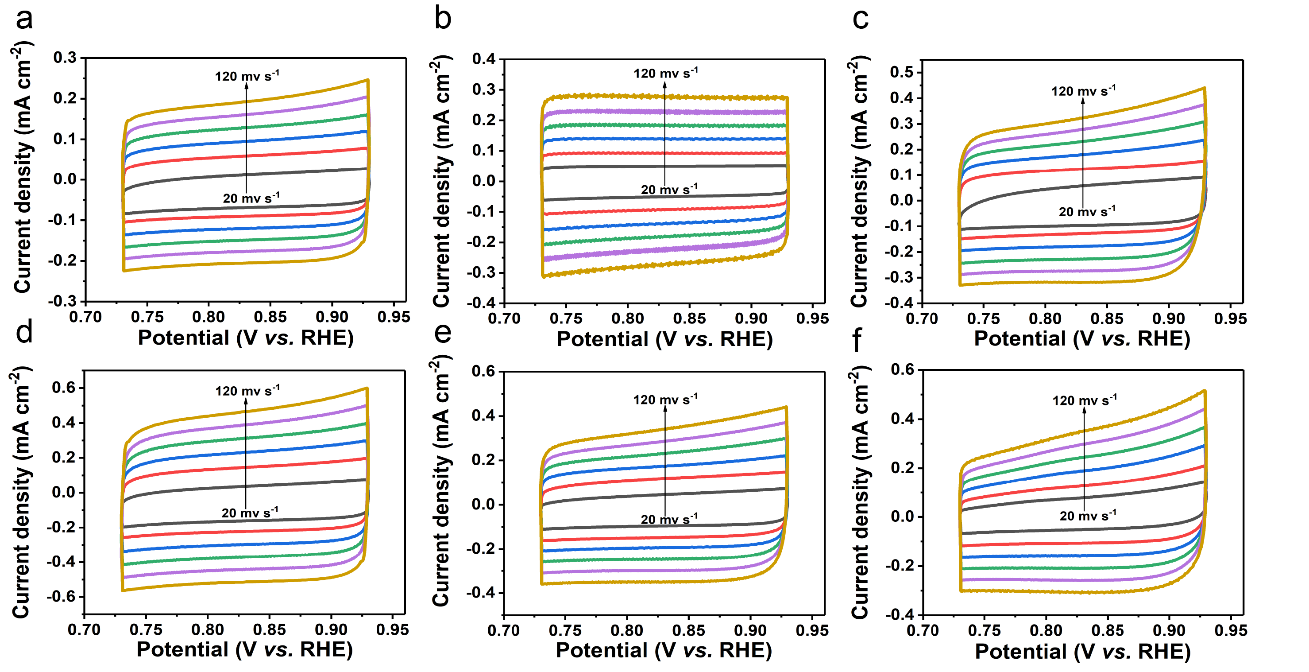


**Fig. S13** Corresponding CV curves withinthe non-Faradic region of **a** NLOS-0@CC, **b** NLOS-0.25@CC, **c** NLOS-0.5@CC, **d** NLOS-1@CC, **e** NLOS-2@CC, and **f** Ni_3_S_2_@CC recorded at the scan rates of 20, 40, 60, 80,100, and 120 mV s^−1^, respectively.

**
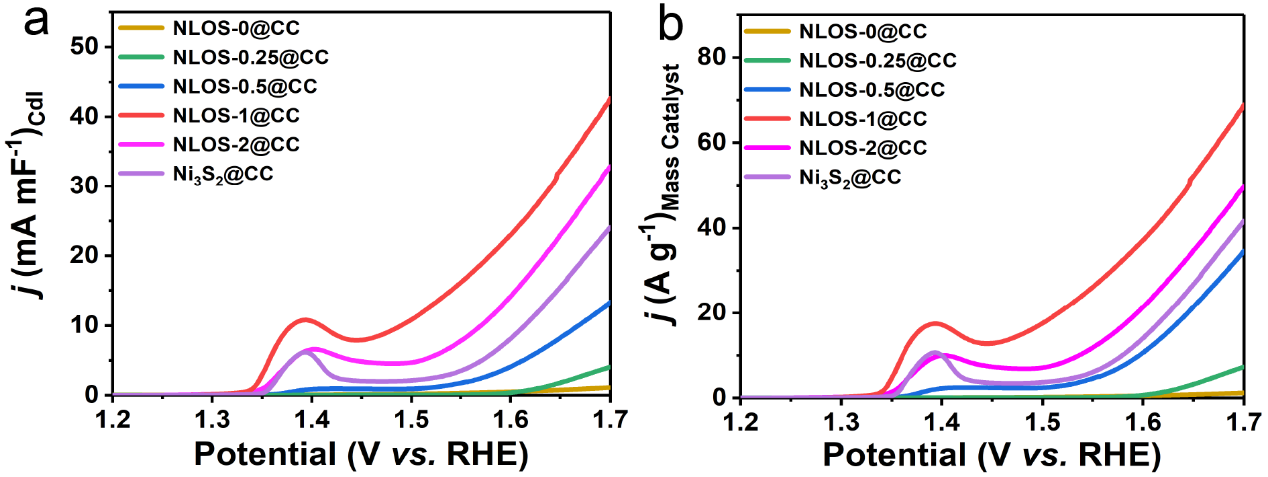
**

**Fig. S14** IR-corrected LSV curves normalized against the **a** *C*_dl_ value, and **b** loading mass of NLOS-0@CC, NLOS-0.25@CC, NLOS-0.5@CC, NLOS-1@CC, NLOS-2@CC, and Ni_3_S_2_@CC. Note that the electrochemical active surface area (ECSA) value is linearly proportional to *C*_dl_ (ECSA=*C*_dl_/*C*_s_), and the *C*_s_ is assumed to be identical for all probed samples in our case. Therefore, we directly normalized our current density to *C*_dl_ values to compare the intrinsic activity of our catalysts [S3, S4].

**

**

**Fig. S15** High-resolution S 2p XPS spectrum of the post-OER NLOS-1@CC.

When compared with the high-resolution S 2p XPS spectrum in Fig. 2f, it can be easily found the concentration ratio between S-O species and M-S within lattice/surface S non-bonded to O increased from 1.79 of the pre-OER NLOS-1@CC to 2.49 of the post-OER one. The presence of S-O in the former case would arise from the surface passivation when exposing the sample to the air, and the increment of S-O species for the same sample after OER could be caused by the presence of the additionally SO_4_^2-^, which was *in-situ* formed and adsorbed on the surface during OER process.

**
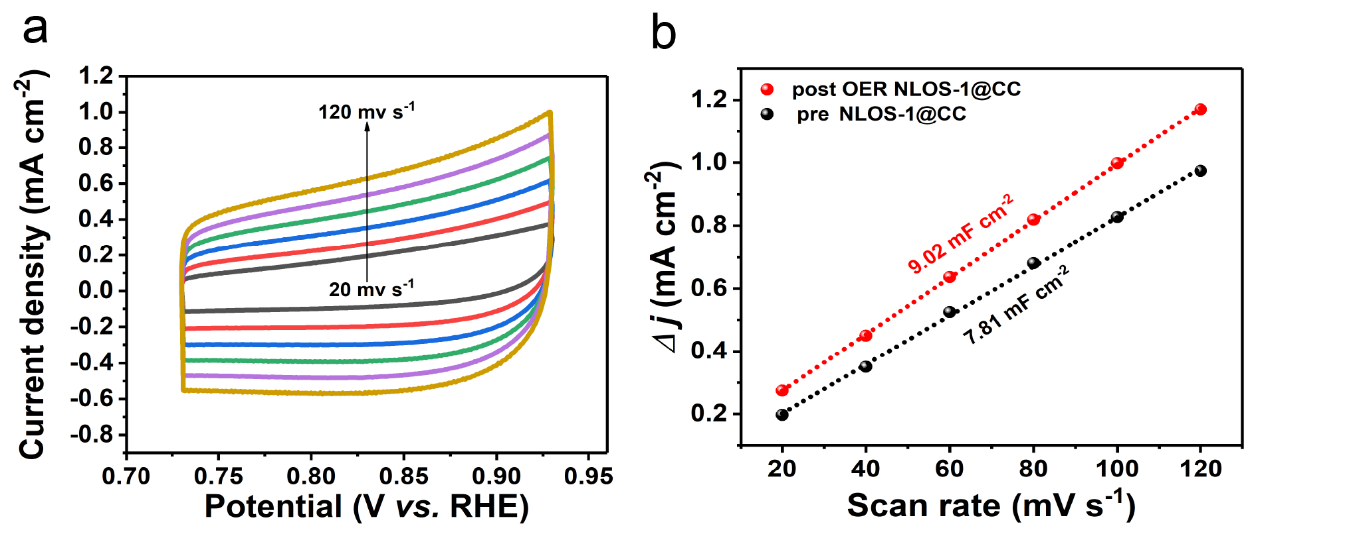
**

**Fig. S16 a** Corresponding CV curves within the non-Faradic region of the post-OER NLOS-1@CC; **b** C_dl_ of pre-OER NLOS-1@CC and post-OER NLOS-1@CC.





**Fig. S17** XRD patterns of the pre- and post-OER NLOS-1@CC. Compared with that of the one before OER, the XRD data of post-OER NLOS-1@CC illustrates a significantly low-crystallinity La_2_O_2_S phase, indicating the occurrence of deep phase reconstruction during alkaline OER.

**
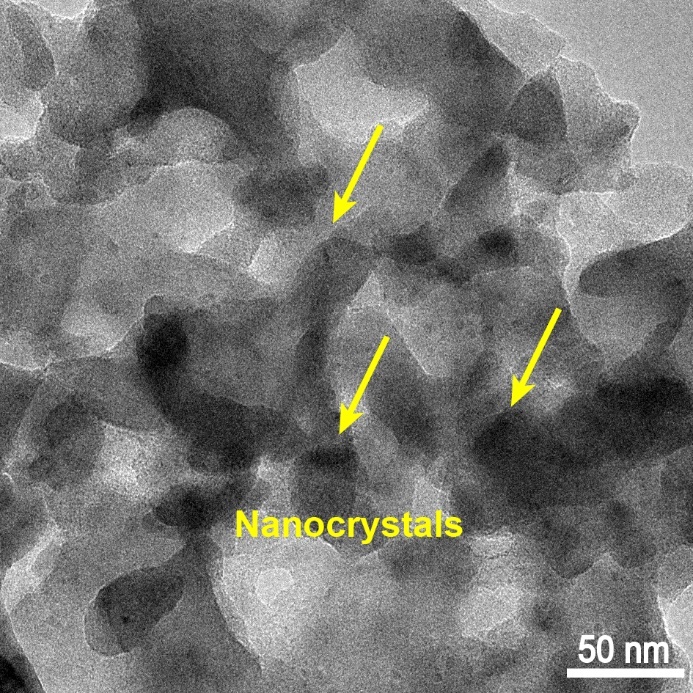
**

**Fig. S18** The magnified HRTEM image of the post-OER NLOS-1, where NiOOH nanocrystals were alternatively separated by La(OH)_3_ nanocrystals.

**

**

**Fig. S19** Time-dependent *in-situ* Raman spectra of NLOS@CC at a fixed potential of 1.7 V *vs.* RHE.

**
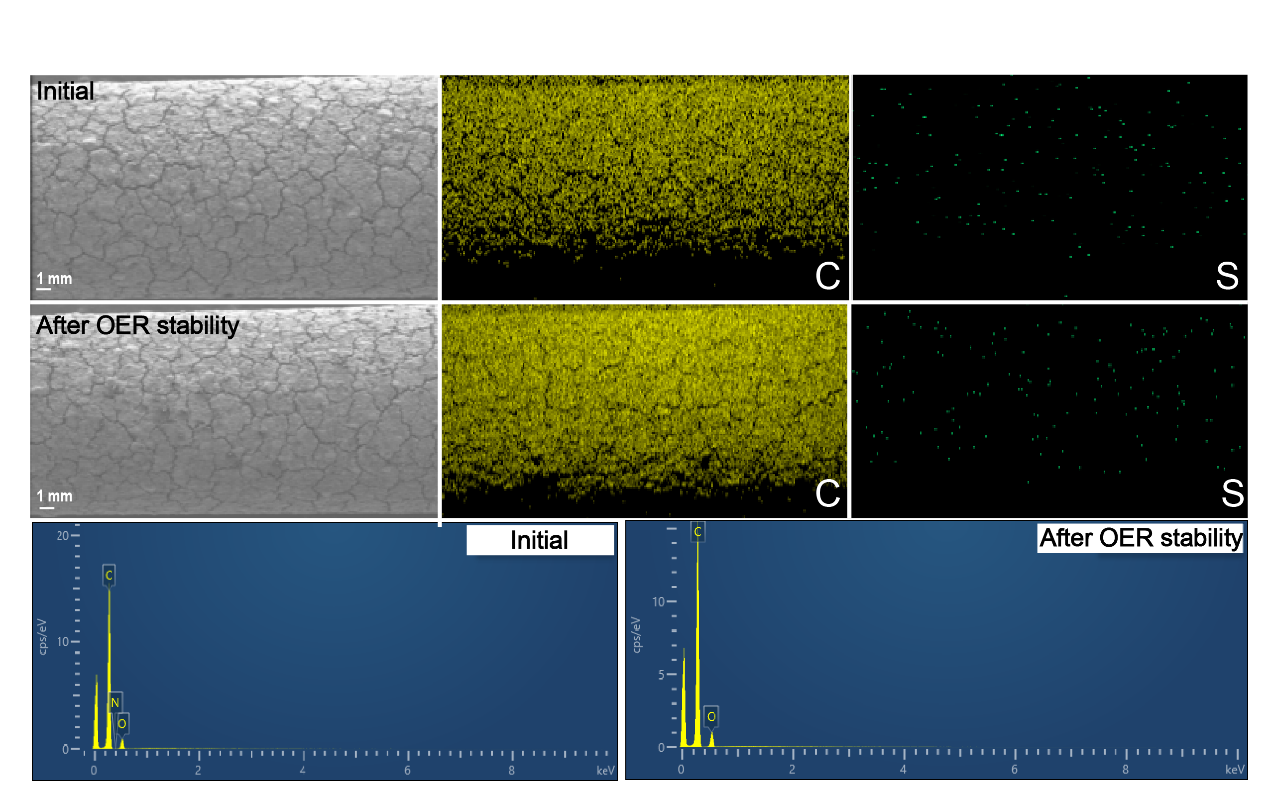
**

**Fig. S20** SEM–EDX and mapping images of the graphite rod before and after OER.

**

**

**Fig. S21** Raman spectrum of the post-OER NLOS-1.

**
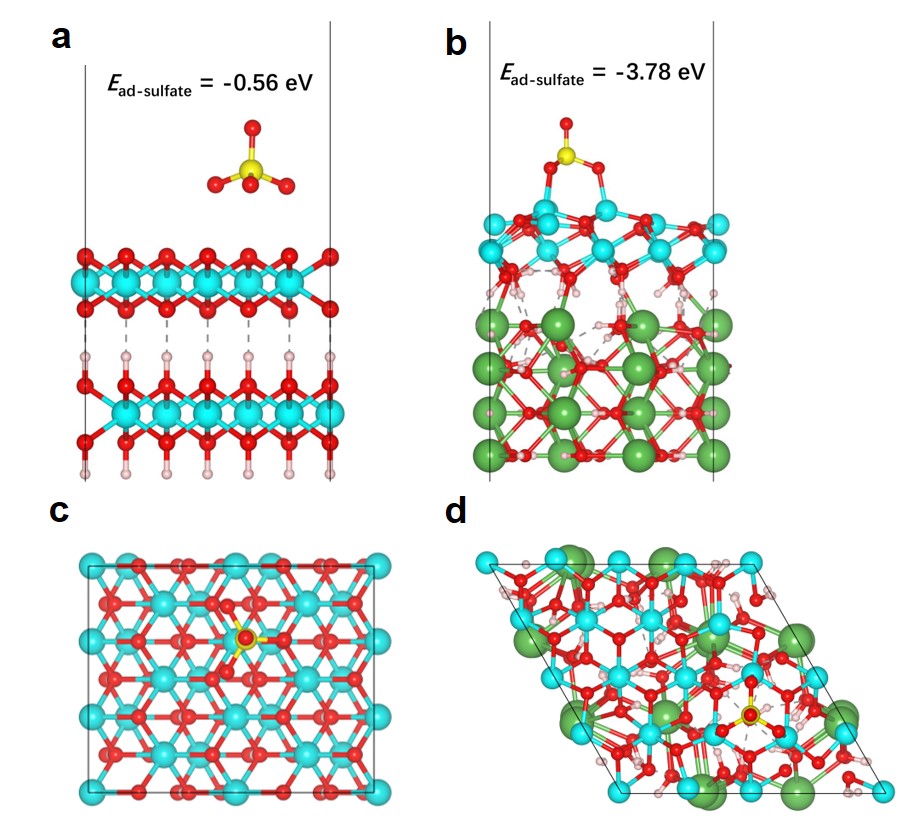
**

**Fig. S22** Comparison of adsorption energy of SO_4_^2-^ (E_ad-sulfate_) on the surface of **a** NiOOH (side view) and **b** NiOOH/La(OH)_3_ (side view). **c** and **d** are their corresponding

crystal structure in top view model.

**Table S1** The atomic ratio of Ni and La of various NLOS-X@CC samples (X=0, 0.25, 0.5, 1, and 2), and Ni_3_S_2_@CC determined by ICP-OES.

| **Samples** | **La** | **Ni** |
| --- | --- | --- |
| NLOS-0@CC | 1 | 0 |
| NLOS-0.25@CC | 1 | 0.45 |
| NLOS-0.5@CC | 1 | 0.62 |
| NLOS-1@CC | 1 | 1.01 |
| NLOS-2@CC | 1 | 2.08 |
| Ni_3_S_2_@CC | 0 | 1 |

**Table S2** Comparison of OER activity of NLOS-1@CC with other recently reported advanced Ni-based electrocatalysts in alkaline media.

| **Catalysts** | **Electrolyte** | **Overpotential**  **(mV) @current density (mA cm^-2^)** | **Ref.** |
| --- | --- | --- | --- |
| Fe, F-NiO | 1 M KOH | 215@10 | [S5] |
| Co_9_S_8_@NiFe LDH  FeNi(MoO_4_)*_x_*  Ni_3_S_2_/MnO_2_ | 1 M KOH  1 M KOH  1 M KOH | 220@10  227@10  260@10 | [S6]  [S7]  [S8] |
| NiO/Co_3_O_4_ | 1 M NaOH | 262@10 | [S9] |
| ex Ir-Ni(OH)_2_ | 1 M KOH | 270@10 | [S10] |
| Ni_0.5_Co_0.5_-MOF-74 | 1 M KOH | 270@10 | [S11] |
| Ni-Fe-K_0.23_MnO_2_ CNFs | 1 M NaOH | 270@10 | [S12] |
| NiCo_2-_*_x_*Fe*_x_*O_4_ NBs | 1 M KOH | 274@10 | [S13] |
| N-NiMoO_4_/NiS_2_ | 1 M KOH | 283@10 | [S14] |
| NiCo_2_S_4_@NiFe | 0.1 M KOH | 287@10 | [S15] |
| NiCeO*_x_* | 1 M KOH | 295@10 | [S16] |
| NiSe_2_/NiO | 1 M KOH | 300@10 | [S17] |
| Mo-Ni-Co-O/Co-N | 1 M KOH | 330@10 | [S18] |
| NP/NiO | 1 M KOH | 332@10 | [S19] |
| NiCo_2_O_4_ | 1 M KOH | 350@10 | [S20] |
| NiFe-V_M_-O | 1 M NaOH | 371@10 | [S21] |
| Fe-VSB/NiPO-500 | 1 M KOH | 227@50 | [S22] |
| NiFe LDH/NiTe | 1 M KOH | 228@50 | [S23] |
| Ni_2_P/FeP-FF | 1 M KOH | 235@50 | [S24] |
| Nb-NiFe-LDH | 1 M KOH | 242@50 | [S25] |
| CoNiFeO*_x_*-NC | 1 M KOH | 265@50 | [S26] |
| Co_0.75_Ni_0.25_Se/NF | 1 M KOH | 269@50 | [S27] |
| S-NiFe-LDH-A | 1 M KOH | 270@50 | [S28] |
| Ni–Fe LDH SSNCs | 1 M KOH | 272@50 | [S29] |
| Ni_2_Fe_1_-O | 1 M KOH | 273@50 | [S30] |
| F-Ni(OH)_2_/NF | 1 M KOH | 280@50 | [S31] |
| np-AlNiCoFeMo | 1 M KOH | 280@50 | [S32] |
| Ni–Fe LDH-NS@DG | 1 M KOH | 310@50 | [S33] |
| Ni(OH)_2_-TCNQ/CF | 1 M KOH | 322@50 | [S34] |
| MoO_3_/Ni–NiO | 1 M KOH | 330@50 | [S35] |
| HG-NiFe*_x_* | 1 M KOH | 350@50 | [S36] |
| **NLOS-1@CC** | **1 M KOH** | **^a^257@10**  **260@50** | **This work** |

^a^This overpotential was obtained from the negatively-scanned LSV curve.

**Table S3** EIS calculation parameters of NLOS-1@CC and reference samples at a catalytically active potential of 1.41V *vs.* RHE in 1 M KOH.

| **Samples** | **R_s_** | **R_ct_** | **CPE1-T** | **CPE2-P** |
| --- | --- | --- | --- | --- |
| NLOS-0@CC | 1.7 | 1204 | 0.0012343 | 0.91912 |
| NLOS-0.25@CC | 1.6 | 378.8 | 0.0021428 | 0.88993 |
| NLOS-0.5@CC | 1.9 | 25.7 | 0.044625 | 0.80676 |
| NLOS-1@CC | 1.6 | 11.1 | 0.10022 | 0.77765 |
| NLOS-2@CC | 1.4 | 25.2 | 0.044316 | 0.80979 |
| Ni_3_S_2_@CC | 1.4 | 53.1 | 0.022986 | 0.85576 |

**Table S4** The atomic ratio of La, Ni, S, and Fe for pre- and post-OER NLOS-1 obtained from TEM-EDS presented in Fig. 3 and Fig. 5 of the main text, respectively.

| **Atomic Ratio** | **La** | **Ni** | **S** | **Fe** |
| --- | --- | --- | --- | --- |
| Theoretic ratio | 1 | 1 | 0.5 | / |
| EDS (pre-OER) | 1 | 1 | 0.49 | / |
| EDS (post-OER) | 0.93 | 1 | 0.12 | 0.006 |

Note after OER CP, there were still a certain amount of S atoms were preserved, which was supposed to contain the ones from the residual NLOS precatalyst, as well as the transformed oxyanions (SO_4_^2-^) adsorbed on the catalyst surface [S37]. Moreover, very few impurity Fe atoms from KOH electrolyte were doped into the reconstructed species, indicating the active structure which was responsible for the excellent OER was Fe-free NiOOH/La(OH)_3_.

**Table S5** The content of La, Ni and S in 1 M KOH electrolyte before and after 72 h OER CP tests of NLOS-1@CC and Ni_3_S_2_@CC.

| **Electrolyte** | **La (mg L**^−1^**)** | **Ni (mg L**^−1^**)** | **S (mg L**^−1^**)** |
| --- | --- | --- | --- |
| Fresh | 0.1 | 0.20 | 1.1 |
| NLOS-1@CC after OER CP | 0.1 | 0.20 | 1.8 |
| Ni_3_S_2_@CC after OER CP | / | 0.37 | 2.2 |

After 72 h OER CP at 100 mA cm^−2^, the contents of La and Ni in the reaction electrolyte were almost the same, while much more S content can be found in the post-OER electrolyte. This illustrates that during OER process, severe S atoms were oxidized from NLOS-1. Apart from some adsorbed on the catalyst surface in the form of SO_4_^2-^, most of the leached S species were dissolved into the electrolyte. On the other hand, when the reaction of Ni_3_S_2_@CC was prolonged to 72 h, much more Ni atoms were lost in the electrolyte, indicating that the presence of La could stabilize the active Ni species and further promoted the catalytic durability.

**Table S6** EXAFS fitting parameters at the La *L_3_*-edge for pre- and post-OER NLOS-1.

| **Samples** | **Shell** | ***CN*** | ***R*(Å)** | ***σ*^2^ (10^–3^ Å^2^)** | ***R* factor** |
| --- | --- | --- | --- | --- | --- |
| Pre-OER | La-O | 4.5±0.3 | 2.38±0.02 | 10.7±1.2 | 0.009 |
|  | La-S | 4.6±0.5 | 3.02±0.01 | 11.2±1.4 |  |
| Post-OER | La-O | 9.8±0.8 | 2.5±0.02 | 8.7±1.1 | 0.016 |
|  | La-S | 3.1±1.7 | 3.1±0.04 | 9.8±5.9 |  |

In Table S6, *CN*, *R*, and *σ*^2^ represent coordination number, distance between absorber and backscatter atoms, respectively. *R* factor reflects the goodness of the fitting.

The obtained EXAFS data were preprocessed using Athena [S38–S40]. Then Fourier transformed fitting was performed by using Artemis. The k-range of 2.6-9.5 Å^−1^ and R range of 1.7-3.0 Å were employed for the fitting. The model of pre- and post-OER NLOS-1 were used to obtain the simulated scattering paths.

**References**

1. F.A. Bushira, P. Wang, Y. Jin, High-entropy oxide for highly efficient luminol–dissolved oxygen electrochemiluminescence and biosensing applications. Anal. Chem. **94**, 2958–2965 (2022). https://doi.org/10.1021/acs.analchem.1c05005
2. Y. Hu, Z. Wu, X. Zheng, N. Lin, Y. Yang, et al., ZnO/ZnGaNO heterostructure with enhanced photocatalytic properties prepared from a LDH precursor using a coprecipitation method. J. Alloys Compd. **709**, 42–53 (2017). https://doi.org/10.1016/j.jallcom.2017.02.124
3. P.W. Menezes, C. Walter, B. Chakraborty, J.N. Hausmann, I. Zaharieva, et al., Combination of highly efficient electrocatalytic water oxidation with selective oxygenation of organic substrates using manganese borophosphates. Adv. Mater. **33**, 2004098 (2021). https://doi.org/10.1002/adma.202004098
4. H. Yang, J.N. Hausmann, V. Hlukhyy, T. Braun, K. Laun, et al., An intermetallic CaFe_6_Ge_6_ approach to unprecedented Ca−Fe−O electrocatalyst for efficient alkaline oxygen evolution reaction. ChemCatChem **14**, e202200293 (2022). <https://doi.org/10.1002/cctc.202200293>
5. C. Lyu, Y. Li, J. Cheng, Y. Yang, K. Wu, et al., Dual atoms (Fe, F) Co-doping inducing electronic structure modulation of NiO hollow flower-spheres for enhanced oxygen evolution/sulfion oxidation reaction performance. Small e2302055 (2023). https://doi.org/10.1002/smll.202302055
6. X.T. Feng, Q.Z. Jiao, Z. Dai, Y.L. Dang, S.L. Suib, et al., Revealing the effect of interfacial electron transfer in heterostructured Co_9_S_8_@NiFe LDH for enhanced electrocatalytic oxygen evolution. J. Mater. Chem. A **9**, 12244–12254 (2021). https://doi.org/10.1039/D1TA02318G
7. K. Dastafkan, S. Wang, C. Rong, Q. Meyer, Y. Li, et al., Cosynergistic molybdate oxo-anionic modification of FeNi-based electrocatalysts for efficient oxygen evolution reaction. Adv. Funct. Mater. **32**, 2107342 (2021). https://doi.org/10.1002/adfm.202107342
8. Y. Xiong, L. Xu, C. Jin, Q. Sun, Interface-engineered atomically thin Ni_3_S_2_/MnO_2_ heterogeneous nanoarrays for efficient overall water splitting in alkaline media. Appl. Catal. B: Environ. **254**, 329–338 (2019). <https://doi.org/10.1016/j.apcatb.2019.05.017>
9. J. Zhang, J. Qian, J. Ran, P. Xi, L. Yang, et al., Engineering lower coordination atoms onto NiO/Co_3_O_4_ heterointerfaces for boosting oxygen evolution reactions. ACS Catal. **10**, 12376–12384 (2020). <https://doi.org/10.1021/acscatal.0c03756>
10. J. Liu, J. Xiao, Z. Wang, H. Yuan, Z. Lu, et al., Structural and electronic engineering of Ir-doped Ni-(oxy)hydroxide nanosheets for enhanced oxygen evolution activity. ACS Catalysis **11**, 5386–5395 (2021). https://doi.org/10.1021/acscatal.1c00110
11. S. Zhao, C. Tan, C.-T. He, P. An, F. Xie, et al., Structural transformation of highly active metal–organic framework electrocatalysts during the oxygen evolution reaction. Nat. Energy **5**, 881–890 (2020). https://doi.org/10.1038/s41560-020-00709-1
12. H. Liao, X. Guo, Y. Hou, H. Liang, Z. Zhou, et al., Construction of defect-rich Ni-Fe-doped K_0.23_MnO_2_ cubic nanoflowers via etching Prussian blue analogue for efficient overall water splitting. Small **16**, 1905223 (2020). <https://doi.org/10.1002/smll.201905223>
13. Y. Huang, S.L. Zhang, X.F. Lu, Z.P. Wu, D. Luan et al., Trimetallic spinel NiCo_2−x_Fe_x_O_4_ nanoboxes for highly efficient electrocatalytic oxygen evolution. Angew. Chem. Int. Ed., **60**, 11841–11846 (2021). https://doi.org/10.1002/ange.202103058
14. L. An, J. Feng, Y. Zhang, R. Wang, H. Liu, et al., Epitaxial heterogeneous interfaces on N-NiMoO_4_/NiS_2_ nanowires/nanosheets to boost hydrogen and oxygen production for overall water splitting. Adv. Funct. Mater. **29**, 1805298 (2019). <https://doi.org/10.1002/adfm.201805298>
15. X. Feng, Q. Jiao, W. Chen, Y. Dang, Z. Dai, et al., Cactus-like NiCo_2_S_4_@ NiFe LDH hollow spheres as an effective oxygen bifunctional electrocatalyst in alkaline solution. Appl. Catal. B: Environ. **286**, 119869 (2021). <https://doi.org/10.1016/j.apcatb.2020.119869>
16. J. Yu, Q. Cao, Y. Li, X. Long, S. Yang, et al. Defect-Rich NiCeOx Electrocatalyst with Ultrahigh Stability and Low Overpotential for Water Oxidation*.* ACS Catal. **9**, 1605–1611 (2019). https://doi.org/10.1021/acscatal.9b00191
17. Z. Liu, C. Zhang, H. Liu, L. Feng, Efficient synergism of NiSe_2_ nanoparticle/NiO nanosheet for energy-relevant water and urea electrocatalysis. Appl. Catal. B: Environ. **276**, 119165 (2020). <https://doi.org/10.1016/j.apcatb.2020.119165>
18. W. Liu, L. Yu, R. Yin, X. Xu, J. Feng, et al., Non-3d metal modulation of a 2D Ni–Co heterostructure array as multifunctional electrocatalyst for portable overall water splitting. Small **16**, 1906775 (2020). <https://doi.org/10.1002/smll.201906775>
19. P. Bhanja, Y. Kim, B. Paul, Y.V. Kaneti, A.A. Alothman, et al., Microporous nickel phosphonate derived heteroatom doped nickel oxide and nickel phosphide: efficient electrocatalysts for oxygen evolution reaction. Chem. Eng. J. **405**, 126803 (2021). <https://doi.org/10.1016/j.cej.2020.126803>
20. L.S. Bezerra, G. Maia, Developing efficient catalysts for the OER and ORR using a combination of Co, Ni, and Pt oxides along with graphene nanoribbons and NiCo_2_O_4_. J. Mater. Chem. A **8**, 17691–17705 (2020). <https://doi.org/10.1039/D0TA05908K>
21. H.J. Lee, S. Back, J.H. Lee, S.H. Choi, Y. Jung et al., Mixed transition metal oxide with vacancy-induced lattice distortion for enhanced catalytic activity of oxygen evolution reaction. ACS Catal. **9**, 7099–7108 (2019). <https://doi.org/10.1021/acscatal.9b01298>
22. J. Zhao, Y. Zhang, H. Guo, H. Zhang, J. Ren et al., Rational regulation of crystalline/amorphous microprisms-nanochannels based on molecular sieve (VSB-5) for electrochemical overall water splitting. Small **18**, e2200832 (2022). https://doi.org/10.1002/smll.202200832
23. L. Hu, X. Zeng, X. Wei, H. Wang, Y. Wu et al., Interface engineering for enhancing electrocatalytic oxygen evolution of NiFe LDH/NiTe heterostructures. Appl. Catal. B Environ., **273**, 1–7 (2020). https://doi.org/10.1016/j.apcatb.2020.119014
24. M. Jiang, H. Zhai, L. Chen, L. Mei, P. Tan et al., Unraveling the synergistic mechanism of Bi-functional nickel–iron phosphides catalysts for overall water splitting. Adv. Funct. Mater. 2302621 (2023). https://doi.org/10.1002/adfm.202302621
25. Y.-N. Zhou, F.-L. Wang, S.-Y. Dou, Z.-N. Shi, B. Dong, et al, Motivating high-valence Nb doping by fast molten salt method for NiFe hydroxides toward efficient oxygen evolution reaction. Chem. Eng. J. **427**, 131643 (2022). https://doi.org/10.1016/j.cej.2021.131643
26. C. Chen, Y. Tuo, Q. Lu, H. Lu, S. Zhang, et al., Hierarchical trimetallic Co-Ni-Fe oxides derived from core-shell structured metal-organic frameworks for highly efficient oxygen evolution reaction. Appl. Catal. B: Environ. **287**, 119953 (2021). https://doi.org/10.1016/j.apcatb.2021.119953
27. S. Liu, Y. Jiang, M. Yang, M. Zhang, Q. Guo, et al., Highly conductive and metallic cobalt–nickel selenide nanorods supported on Ni foam as an efficient electrocatalyst for alkaline water splitting. Nanoscale **11**, 7959–7966 (2019). https://doi.org/10.1039/C8NR10545F
28. Y.-N. Zhou, W.-L. Yu, Y.-N. Cao, J. Zhao, B. Dong, et al., S-doped nickel-iron hydroxides synthesized by room-temperature electrochemical activation for efficient oxygen evolution. Appl. Catal. B: Environ. **292**, 120150 (2021). https://doi.org/10.1016/j.apcatb.2021.120150
29. J. Zhang, L. Yu, Y. Chen, X.F. Lu, S. Gao et al., Designed formation of double-shelled Ni–Fe layered-double-hydroxide nanocages for efficient oxygen evolution reaction. Adv. Mater. **32**, 1906432 (2020). https://doi.org/10.1002/adma.201906432
30. C. Dong, T. Kou, H. Gao, Z. Peng, Z. Zhang, Eutectic-derived mesoporous Ni-Fe-O nanowire network vatalyzing oxygen evolution and overall water splitting. Adv. Energy Mater. **25**, 1701347 (2018). https://doi.org/10.1002/aenm.201701347
31. S.J. Patil, N.R. Chodankar, S.K. Hwang, G.S.R. Raju, Y.S. Huh et al., Fluorine engineered self-supported ultrathin 2D nickel hydroxide nanosheets as highly robust and stable bifunctional electrocatalysts for oxygen evolution and urea oxidation reactions. Small **18**, 2103326 (2022). https://doi.org/10.1002/smll.202103326
32. H.-J. Qiu, G. Fang, J. Gao, Y. Wen, J. Lv, et al., Noble metal-gree nanoporous high-entropy alloys as highly efficient electrocatalysts for oxygen evolution reaction. ACS Materials Lett. **1**, 526–533 (2019). https://doi.org/10.1021/acsmaterialslett.9b00414
33. Y. Jia, L. Zhang, G. Gao, H. Chen, B. Wang, et al., A heterostructure coupling of exfoliated Ni–Fe hydroxide nanosheet and defective graphene as a bifunctional electrocatalyst for overall water splitting. Adv. Mater. **29**, 1700017 (2017). <https://doi.org/10.1002/adma.201700017>
34. X. Guo, R. Kong, X. Zhang, H. Du, F. Qu, Ni(OH)_2_ nanoparticles embedded in conductive microrod array: An efficient and durable electrocatalyst for alkaline oxygen evolution reaction. ACS Catal. **8**, 651–655 (2018). <https://doi.org/10.1021/acscatal.7b03406>
35. X. Li, Y. Wang, J. Wang, Y. Da, J. Zhang, et al., Sequential electrodeposition of bifunctional catalytically active structures in MoO_3_/Ni–NiO composite electrocatalysts for selective hydrogen and oxygen evolution. Adv. Mater. **32**, 2003414 (2020). https://doi.org/10.1002/adma.202003414
36. J. Wang, L.Y. Gan, W.Y. Zhang, Y.C. Peng, H. Yu, et al., In situ formation of molecular Ni-Fe active sites on heteroatom-doped graphene as a heterogeneous electrocatalyst toward oxygen evolution. Sci. Adv. **4**, 7970–7977 (2018). https://doi.org/10.1126/sciadv.aap7970
37. J.N. Hausmann, P.W. Menezes, Effect of surface-adsorbed and intercalated (oxy) anions on the oxygen evolution reaction. Angew. Chem. Int. Ed. **61**, e202207279 (2022). https://doi.org/10.1002/anie.202207279
38. H. Funke, A.C. Scheinost, M. Chukalina, Wavelet analysis of extended x-ray absorption fine structure data. Phys. Rev. B **71**, 94110 (2005). https://doi.org/10.1103/PhysRevB.71.094110
39. B. Ravel, M. Newville, ATHENA, ARTEMIS, HEPHAESTUS: data analysis for X-ray absorption spectroscopy using IFEFFIT. J. Synchrotron Radiat. **12**, 537–541 (2005). https://doi.org/10.1107/S0909049505012719
40. H. Funke, M. Chukalina, A.C. Scheinost, A new FEFF-based wavelet for EXAFS data analysis. J. Synchrotron Radiat. **14**, 426–432 (2007). <https://doi.org/10.1107/S0909049507031901>
